# Determinants of prolonged hospitalization following shoulder arthroplasty: a systematic review of patient and health system factors

**DOI:** 10.1016/j.xrrt.2026.100856

**Published:** 2026-08-26

**Authors:** Hussayn Shinwari, Hamzah Chaudhry, Bilal Ceesay, Hanan Taimur Shinwari, Wisal Khan, Usman Tahir, Arnau Sheikh, Jawwad Mihran Haider, Abith Ganesh Kamath, Saran Singh Gill, Kapil Sugand

**Affiliations:** a Worcestershire Acute Hospitals NHS Trust, Worcestershire Royal Hospital, Worcester, UK; b Faculty of Medicine, St George's University of London, London, UK; c Faculty of Medicine, University of Sheffield, Sheffield, UK; d Institute of Biotechnology and Genetic Engineering (IBGE), The University of Agriculture Peshawar, Peshawar, Pakistan; e Barts Health NHS Trust, The Royal London Hospital, London, UK; f St George’s University Hospitals NHS Foundation Trust, St George’s Hospital, London, UK; g Faculty of Medicine, Imperial College London, London, UK

**Keywords:** Shoulder arthroplasty, Length of stay, Prolonged hospitalization, Predictors, Risk factors, Same-day discharge, Patient factors, Health system factors

## Abstract

**Background:**

Shoulder arthroplasty utilization is rising rapidly worldwide, driven predominantly by reverse procedures, with volumes projected to exceed 350,000 annually in the United States by 2025. Hospital length of stay (LOS) is a key driver of cost and resource utilization, yet no comprehensive synthesis of patient- and health system-related predictors exists.

**Methods:**

This systematic review adhered to Preferred Reporting Items for Systematic Reviews and Meta-Analyses 2020 and Synthesis Without Meta-analysis guidelines. Five databases (PubMed, Embase, Medline, Scopus, and Web of Science) were searched from inception to July 2025. Eligible studies assessed factors influencing LOS or prolonged hospitalization following shoulder arthroplasty. Qualitative synthesis was conducted due to heterogeneity.

**Results:**

A total of 52 studies comprising 2,153,034 patients were included. Advancing age (commonly ≥75-80 years; LOS increase 0.4-1.8 days; odds ratio [OR] 1.8-3.6) and female sex (OR: 1.5-2.8) were the most consistent predictors of prolonged LOS. Morbid obesity (body mass index ≥40 kg/m^2^; LOS increase 0.3-0.9 days), non-White ethnicity (particularly Black patients; LOS increase 0.1-0.5 days), adverse social determinants (eg, living alone; OR: 1.4-1.6), public insurance (Medicaid/Medicare; OR: 1.6-4.8), and low hospital or surgeon volume (LOS increase 0.4-0.9 days) were also independently associated with longer stays.

**Conclusion:**

Prolonged LOS after shoulder arthroplasty is multifactorial, with non-modifiable demographic factors compounded by modifiable social and structural elements. These findings provide an evidence-based framework for risk stratification, pre-operative optimization, and health system redesign to facilitate safe LOS reduction and equitable care amid escalating procedural demand.

Shoulder arthroplasty (SA) utilization has risen worldwide owing to expanding indications and improved implant designs, with substantial improvements in patient outcomes.^17^^,^^33^^,^^43^ Between 2011 and 2017, primary SA volume in the United States more than doubled from 51,329 to 104,575 procedures and is projected to rise an additional 235% to 350,558 procedures by 2025, far outpacing the projected growth of total hip (47%) and knee arthroplasty (22%) over the same period.^67^ This rising procedural volume increases demand on hospital resources, including inpatient beds and discharge pathways.

Hospital length of stay (LOS) is a major determinant of cost and resource utilization after SA. Although mean LOS has decreased from 2.2 ± 1.7 days (2005-2010) to 1.7 ± 3.6 days (2015-2018), patients undergoing surgery today present with greater burdens of comorbidity and more complex perioperative needs.^6^ Evidence suggests carefully selected patients can safely undergo same-day discharge with low complication and readmission rates.^11^^,^^19^^,^^44^^,^^64^ These findings suggest prolonged LOS reflects both patient- and health system-level factors, highlighting opportunities for targeted optimization.

While individual observational studies have explored determinants of LOS after SA, there is currently no comprehensive systematic synthesis of these predictors. Therefore, this systematic review aims to synthesize all available evidence on patient- and health system-related factors associated with hospital LOS and prolonged hospitalization following SA, identify consistent predictors across studies, and inform strategies for improved patient selection, perioperative optimization, and healthcare resource planning.

## Methodology

### Adherence to guidelines

The systematic review was conducted in line with the Preferred Reporting Items for Systematic Reviews and Meta-Analyses 2020 guidelines.^46^ The completed Preferred Reporting Items for Systematic Reviews and Meta-Analyses flowchart was shown in Figure 1. Analysis was conducted in line with Synthesis Without Meta-analysis guidelines. The review was registered with International Prospective Register of Systematic Reviews (CRD420251276671).^10^

**Figure 1:**
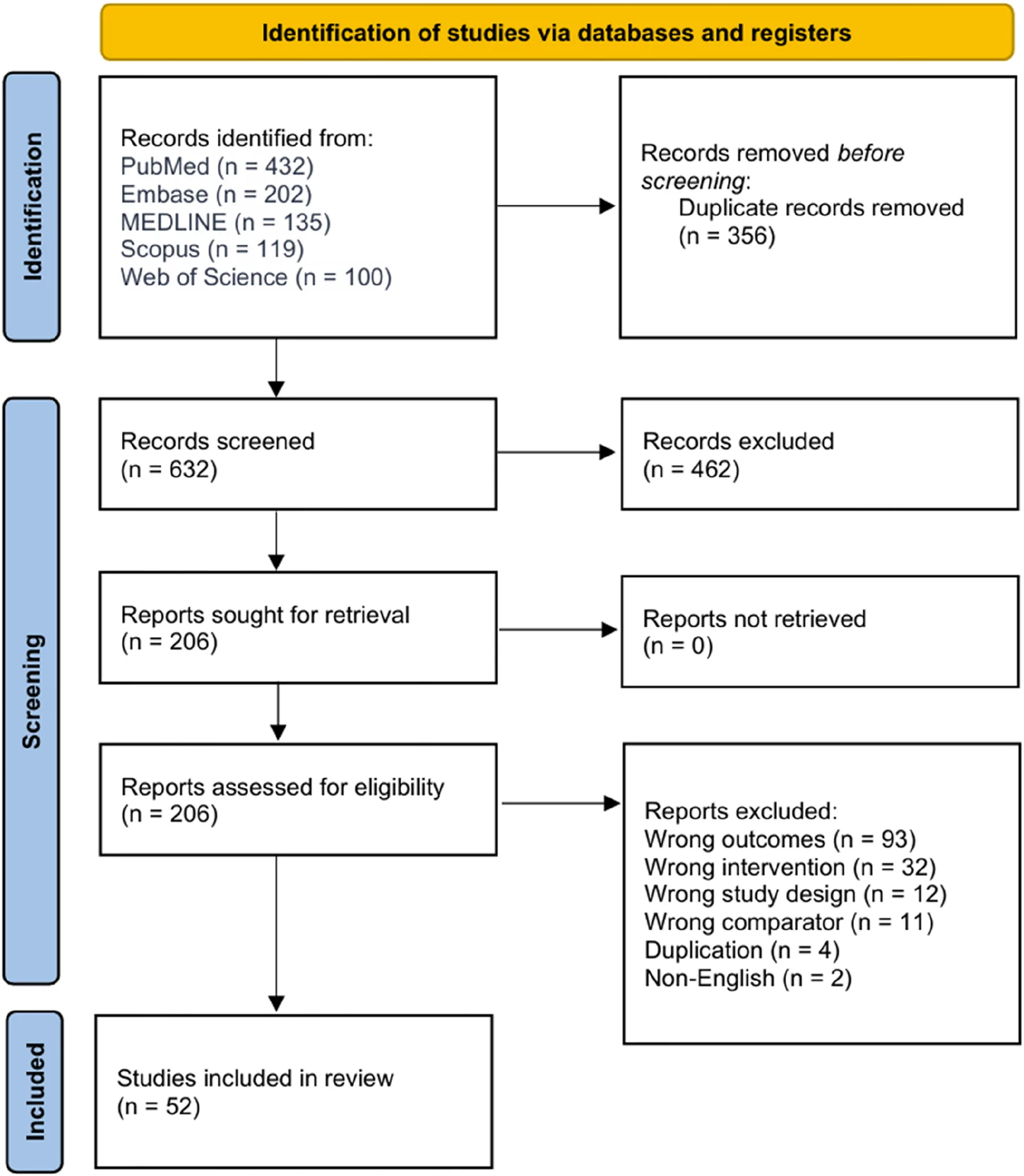
PRISMA flow diagram. *PRISMA*, Preferred Reporting Items for Systematic Reviews and Meta-Analyses.

### Electronic databases

A multi-database search was performed from conception to July 20, 2025, encompassing PubMed, Embase, Medline, Scopus, and Web of Science. Our full search string can be found in Supplementary Table S1.

### Eligibility criteria

Eligible studies included adult patients (≥18 years) undergoing primary or revision shoulder arthroplasty (total shoulder arthroplasty [TSA], reverse shoulder arthroplasty [rTSA], or hemiarthroplasty) that assessed patient- or health system-related factors influencing hospital LOS, prolonged hospitalization, or failure of same-day discharge. Studies were required to include a comparator or reference group. Observational studies and registry/database analyses were included. Studies focusing on pediatric patients, non-arthroplasty procedures, interventions unrelated to LOS, or lacking LOS data or comparators were excluded. Only English-language full-text articles were considered. Supplementary Table S2 outlines the Population, Intervention, Comparison, and Outcomes (PICO) criteria.

### Screening and data extraction

Studies were imported into Covidence software, where duplicates were removed. Three reviewers (H.S., H.C., and B.C.) initially screened titles and abstracts, following Cochrane Handbook recommendations to retain potentially relevant studies. Full-text articles were reviewed for eligibility based on the PICO framework.^14^^,^^52^ Data extraction was performed independently by 3 reviewers (H.S., H.C., B.C.). Discrepancies were resolved by discussion, with final arbitration by the senior author (K.S.). Data were extracted using an Excel (Microsoft Inc., Washington, USA) spreadsheet including study characteristics, study design, patient demographics, location, publication year, sample size, and outcomes assessed.

### Risk of bias

Two independent reviewers (H.C. and B.C.) assessed the risk of bias using Risk Of Bias In Non-randomized Studies - of Interventions (ROBINS-I) for non-randomized studies as shown in Figure 2.^65^ Disagreements were resolved through discussion, with a third reviewer (H.S.) providing arbitration when necessary. The risk of bias assessment focused on domains like randomization, blinding, and handling of missing data to ensure result validity.

**Figure 2:**
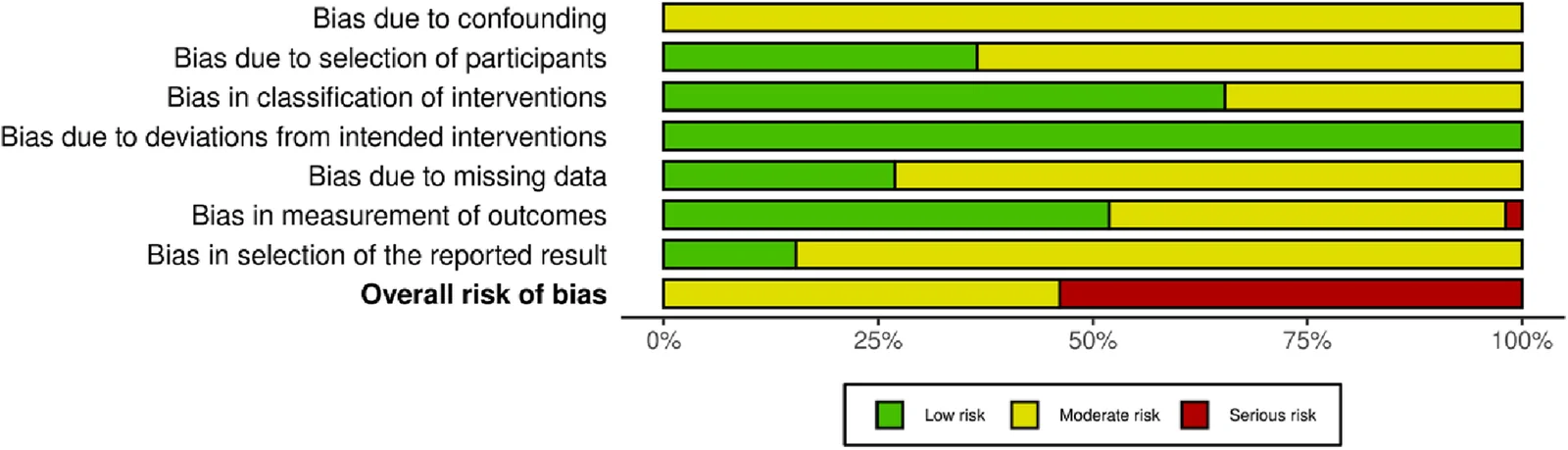
ROBINS-I risk of bias. *ROBINS-I*, Risk Of Bias In Non-randomized Studies - of Interventions.

### Data analysis, qualitative synthesis, and reporting

Due to substantial heterogeneity across studies in arthroplasty type (anatomic versus reverse), surgical indications, perioperative protocols, definitions of LOS (continuous versus dichotomous), and recurring administrative databases representing distinct study populations and enrollment periods, quantitative meta-analysis was not feasible. Instead, a qualitative synthesis was conducted as per Synthesis Without Meta-analysis guidance.^10^

## Results

### Study selection

A total of 988 articles were initially identified (Fig. 1). After removal of duplicates and detailed screening against predefined inclusion and exclusion criteria, 52 studies involving a total of 2,153,034 patients were deemed eligible and included in the final analysis (1,2,4,6–8,11,12,14,15,17,19–31,33–41,45–49,51–60,63,65–67).

### Study characteristics

The mean age of patients across studies ranged from 57 to 84 years. Gender representation varied widely, with the proportion of males ranging from 16% to 53.8% across studies. Mean body mass index (BMI), where reported, ranged from 26.8 to 43.2 kg/m^2^. Sample sizes differed substantially, from as few as 29 patients to over 349,046 (Table I, Table II, Table III).

**Table I:** Patient demographics.

Author (yr)	Title	Journal	Study design	Country or region	Sample size (total and for each subgroup (if applicable)	Age (mean ± SD or median [IQR])	% male	BMI (mean ± SD)	Comorbidity indices	Indication for TSA	Type of TSA performed
Nazzal^41^ 2025	Predictors of failing same-day discharge after shoulder arthroplasty: developing a model to improve outcomes and reduce health care cost.	*Journal of Shoulder and Elbow Surgery*	Retrospective case-control study	United States	333 patients after exclusions; 69 anatomic TSA (aTSA) and 264 reverse TSA (rTSA) 241 patients (72.4%) achieved same day discharge (SDD) while 92 (27.6%) failed SDD and required an overnight stay.	69.9 ± 7.9 yr overall. Achieved SDD had a mean age of 69.5 ± 7.7 vs. 71.1 ± 8.2 yr in patients who failed SDD.	46.9% male overall; 50.2% male in the SDD group vs. 38.0% male in the failure group.	Mean BMI 30.9 ± 6.4 kg/m^2^; SDD group 30.5 ± 6.4 vs. failure group 32.0 ± 6.3.	Mean Charlson Deyo Comorbidity Index (CCI) 3.4 ± 1.6 overall (3.33 ± 1.6 for SDD vs. 3.67 ± 1.7 for failure)	Primary glenohumeral osteoarthritis or rotator cuff arthropathy. Patients with fractures, revision arthroplasty, or age <45 yr were excluded.	Anatomic TSA, reverse TSA
Areti^2^ 2025	Impact of morbid obesity on postoperative outcomes in reverse total shoulder arthroplasty: A national inpatient sample analysis.	*Journal of Orthopaedics*	Retrospective cohort study	United States	59,925 total patients: 4,850 morbidly obese patients (morbid-obesity (+) group) and 55,075 non-morbidly obese patients (control group).	Mean age at admission: morbidly obese 67.74 ± 7.674 yr vs. 71.67 ± 8.642 yr in the control group (difference −3.93 yr, *P* < .001). Age categories showed a greater proportion of younger patients in the morbidly obese cohort (<60 yr: 14.15% vs. 8.32% control; 60–69 yr: 42.55% vs. 29.84%; *P* < .001).	28.40% in morbidly obese vs. 40.44% male control group.		Diabetes and tobacco-related disorders. Diabetes without complications occurred in 22.04% of morbidly obese vs. 13.78% controls; diabetes with complications occurred in 0.39% vs. 0.18%. Tobacco-related diagnoses were less common in the morbidly obese group (11.26% vs. 16.51%, *P* < .001).	Rotator cuff arthropathy, fractures, osteoarthritis, and revision arthroplasty	Reverse total shoulder arthroplasty only
Burns^9^ 2024	Prior nonarthroplasty shoulder surgery and modifiable risk factors negatively affect patient outcomes after shoulder arthroplasty.	*JSES International*	Retrospective cohort study	United States	512 patients	Mean age 70.7 ± 9.1 yr in the no-prior-surgery group vs. 65.2 ± 9.3 yr in the prior-surgery group (*P* < .001)	40.3% male in the no-prior-surgery cohort vs. 52.2% male in the prior-surgery cohort (*P* = .016)	Mean BMI 31.7 ± 6.8 kg/m^2^ (no prior surgery) vs. 31.4 ± 6.3 kg/m^2^ (prior surgery)	ASA classification used - modifiable risk factors included smoking, opioid use, uncontrolled diabetes, obesity, anemia, and malnutrition	Primary shoulder arthroplasty (anatomic or reverse) for various degenerative or rotator-cuff–related conditions	Both total shoulder arthroplasty and reverse total shoulder arthroplasty
Gammel^18^ 2025	Medicaid status is independently predictive of increased complications, readmission, and mortality following primary total shoulder arthroplasty.	*Journal of Shoulder and Elbow Surgery*	Retrospective cohort study	United States	Total n = 30,822 cases 15,448 Medicaid and 15,374 control patients	57 yr	43% male		Charles Deyo Comorbidity Index (CCI). Medicaid patients had a higher mean CCI (1.2 ± 1.7) than controls (0.8 ± 1.4). They also had higher rates of congestive heart failure, chronic lung disease, coagulopathy, deficiency anemia, dementia, fluid/electrolyte disorders, hypertension, liver disease, lymphoma, myocardial infarction, paralysis, peptic ulcer disease, renal failure, and substance abuse.	Elective primary shoulder arthroplasty	Anatomic and reverse total shoulder arthroplasty
Sohn^63^ 2024	The Influence of Solid Organ Transplant on Inpatient Complications, Length of Stay, and Hospital Costs in Reverse Shoulder Arthroplasty Patients.	*Cureus*	Retrospective cohort study	United States	59,925 rTSA patients were identified; 59,769 (99.7%). 156 patients remained in the SOT cohort and 156 in the matched control cohort	Unmatched cohort: mean age 67.0 ± 7.1 yr in the SOT group vs. 71.4 ± 8.6 yr in the non-SOT group (*P* < .001)	Unmatched cohort: 53.8% male in the SOT group vs. 39.3% male in the non-SOT group (*P* < .001)		Prevalence in unmatched cohort: diabetes 15.3% (SOT) vs. 14.4% (non-SOT); tobacco use disorder 12.8% vs. 16.1%; obesity 14.7% vs. 19.9%	Reverse shoulder arthroplasty	Reverse shoulder arthroplasty
Raji^50^ 2024	Same-day discharge vs. inpatient total shoulder arthroplasty: an age stratified comparison of postoperative outcomes and hospital charges.	*Journal of Shoulder and Elbow Surgery*	Retrospective cohort study	United States	295 primary TSA cases. After applying SDD vs. IP definition, 121 patients were treated as SDD and 174 as IP.	Mean age: SDD 66.43 ± 9.71 yr; IP 70.83 ± 10.19 yr (*P* < .001).	SDD 50.41% male vs. IP 32.76% male same-day discharge (SDD; length of stay ≤8 h) or inpatient (IP; LOS >8 h)		Elixhauser Comorbidity Index (ECI) was 0 (IQR –3 to 5) for SDD and 2 (−1 to 5) for IP (*P* = .067). Prevalences of diabetes (14.9% vs. 19.5%), hypertension (69.4% vs. 78.2%), cardiac disease (23.1% vs. 23.6%), pulmonary disease (25.6% vs. 25.9%), chronic kidney disease (16.5% vs. 21.8%), and malignancy history (13.2% each) were comparable between SDD and IP cohorts.	Primary TSA for glenohumeral osteoarthritis, cuff tear arthropathy or avascular necrosis	Anatomic vs. reverse TSA. SDD: 52.89% anatomic and 47.11% reverse; IP: 40.23% anatomic and 59.77% reverse.
Mayfield^37^ 2024	Shoulder arthroplasty for inflammatory arthritis is associated with higher rates of medical and surgical complications: a nationwide matched cohort analysis from 2016-2020.	*JSES International*	Retrospective database cohort study	United States	Total post-match (final analysis): IA 4,082, OA 32,656. aTSA (post-match): IA 1,343, OA 10,744. rTSA (post-match): IA 2,739, OA 21,912.	Post-match overall: IA 70.39 ± 8.01 vs. OA 70.46 ± 8.20. aTSA: IA 66.91 ± 8.31 vs. OA 66.94 ± 8.51. rTSA: IA 72.09 ± 7.28 vs. OA 72.20 ± 7.46.	Post-match 31.99%		Individual comorbidities (eg, HTN, COPD, renal failure, depression, coagulation disorder, iron-deficiency anemia, fluid/electrolyte imbalance) compared pre-/post-match	Inflammatory arthritis vs. osteoarthritis	Anatomic TSA, reverse TSA
Gruson^22^ 2023	Does Morbid Obesity (BMI ≥40 kg/m2) Impact Operative Time, Blood Loss, Length of Stay, or Complications Following Anatomic Total Shoulder Arthroplasty?	*Archives of Bone and Joint Surgery*	Retrospective cohort study	United States	Total n = 130: non-obese (<30 kg/m^2^) n = 47, obese (30–39.9 kg/m^2^) n = 60, morbidly obese (≥40 kg/m^2^) n = 23	Median ages: non-obese 64.0 yr (IQR 56–71), obese 61.5 yr (IQR 55–69), morbidly obese 62.0 yr (IQR 56–67)	42% (55/130)	Median BMI: non-obese 26.8 kg/m^2^ (IQR 24.9–28.2), obese 33.9 kg/m^2^ (IQR 32.4–36.5), morbidly obese 42.7 kg/m^2^ (IQR 41.3–45.4). Overall mean BMI 33.2 ± 6.8 kg/m^2^	Age-adjusted Charlson Comorbidity Index (ACCI); medians: 3.0 (IQR 2.0–4.0) non-obese, 2.5 (IQR 1.5–4.0) obese, 2.0 (IQR 2.0–3.0) morbidly obese	Elective primary aTSA for primary osteoarthritis	Anatomic total shoulder arthroplasty
Puzzitiello^49^ 2022	Patients with limited health literacy have worse preoperative function and pain control and experience prolonged hospitalizations following shoulder arthroplasty.	*Journal of Shoulder and Elbow Ssurgery*	Retrospective cohort study	United States	Total = 230 patients. Limited health literacy (LHL) cohort: 58 patients (25.2%). High health literacy (HHL) cohort: 172 patients (74.8%).	Mean age: HHL 67.9 ± 7.7 yr vs. LHL 69.0 ± 9.6 yr (*P* = .36).	HHL 50% vs. LHL 51.7% (*P* = .82)	HHL 30.8 ± 6.4 kg/m^2^ vs. LHL 31.6 ± 6.9 kg/m^2^ (*P* = .42)	ASA physical status classification (ASA >2) and Area Deprivation Index (ADI)	Osteoarthritis or rotator cuff arthropathy	Reverse TSA and anatomic TSA
Burns^8^ 2022	Modifiable risk factors increase length of stay and 90-day cost of care after shoulder arthroplasty.	*JSES*	Retrospective cohort study	United States	1,317				Modifiable risk factors (MRFs) defined as anemia (hemoglobin <10 g/dL), malnutrition (albumin <3.4 g/dL), obesity (BMI >40 kg/m^2^), uncontrolled diabetes (random glucose >180 mg/dL or HbA1c > 8%), tobacco use, and opioid use.	Acute fracture	
Lunati^34^ 2021	Preoperative depression is a risk factor for complication and increased health care utilization following total shoulder arthroplasty.	*JSES*	Retrospective cohort study	United States	Total n = 22,623 non-depression cohort n = 19,414; depression cohort n = 3,209		Overall ≈49% male (52.1% male in non-depressed vs. 32.4% in depressed cohort)		Elixhauser method: obesity, chronic kidney disease, alcohol use, tobacco use, hypertension, coronary artery disease, anxiety, congestive heart failure, rheumatic disease, and diabetes	Primary TSA for glenohumeral disease	Anatomic (TSA) and reverse (rTSA)
Polisetty^48^ 2021	Determining the validity of the Outpatient Arthroplasty Risk Assessment (OARA) tool for identifying patients for safe same-day discharge after primary shoulder arthroplasty.	*JSES*	Retrospective review	United States	422	71.6 ± 8.3 yr	48.30%	28.5 ± 6.0 kg/m^2^	OARA score	Primary elective shoulder arthroplasty	Anatomic and reverse TSA
Dacombe^13^ 2020	Predictors of length of stay following shoulder arthroplasty in a high-volume UK centre.	*Annals of the Royal College of Surgeons of England*	Retrospective consecutive cohort study	UK	566 patients	Overall mean age 72 ± 10 yr	29.50%		Charlson Comorbidity Index, ASA distribution	Osteoarthritis (297 cases), cuff tear arthropathy (160), rheumatoid arthritis (36), avascular necrosis (18), trauma (22), trauma sequelae (49), infection (3), revision (55)	Anatomic total shoulder replacement (262), reverse shoulder arthroplasty (287), hemiarthroplasty (36), revision arthroplasty (55)
Menendez^38^ 2019	Delayed hospital discharge after total shoulder arthroplasty: why, and who is at risk?	*JSES Open Access*	Retrospective cohort study	United States	415 patients	68.8 ± 8.4 yr	39%	30.8 ± 6.3 kg/m^2^	ASA class distribution, depression, diabetes	Elective primary anatomic or reverse TSA for degenerative shoulder conditions	Anatomic (TSA) and reverse (rTSA)
Biron^5^ 2020	A Novel Machine Learning Model Developed to Assist in Patient Selection for Outpatient Total Shoulder Arthroplasty.	*J Am Acad Orthop Surg*	Retrospective cross-sectional analysis	United States	4,500	67.8 yr in short-stay vs. 72.8 yr in long-stay	51.1% male (1,084/2,122); long-stay: 31.9% male (321/1,006)	BMI 31.2 kg/m^2^ in short-stay vs. 31.7 kg/m^2^ long stay	ASA class, diabetes, CHF, COPD, hypertension, dyspnea, obesity	Elective total shoulder arthroplasty	Anatomic and reverse TSA
Singh^58^ 2020	Insurance Payer Type and Patient Income Are Associated with Outcomes after Total Shoulder Arthroplasty.	*Journal of Rheumatology*	Cross-sectional analysis	United States	349,046	Median 68.6 yr (IQR 13.8)	45.90%		Charlson Deyo Comorbidity Score	Osteoarthritis (82.6%), rheumatoid arthritis (1.6%), aseptic bone necrosis (1.9%), fracture (2.5%), other diagnoses (11.4%)	
Navarro^40^ 2019	Short-term outcomes after anatomic total shoulder arthroplasty in patients with osteoarthritis versus osteonecrosis.	*Annals of Translational Medicine*	Retrospective cohort study	United States	516	Mean age: OA 63 yr; ON 62 yr	OA cohort 18.3% male; ON cohort 22.5% male	Mean BMI 30 kg/m^2^	Charlson/Deyo score	Primary glenohumeral osteoarthritis (OA) or osteonecrosis (ON)	Anatomic total shoulder arthroplasty
Sabesan^53^ 2020	Relationship between hospital size and teaching status on outcomes for reverse shoulder arthroplasty.	*Musculoskeletal*	Retrospective cohort study	United States	24,056	72.6 yr	36%		Baseline comorbidities (eg, hypertension, chronic pulmonary disease, diabetes)	Reverse shoulder arthroplasty	Reverse shoulder arthroplasty
Crook^12^ 2018	Comparison of 90-Day Perioperative Outcomes in Shoulder Arthroplasty Between the Elderly and Nonelderly Patients.	*Geriatric and Orthopaedic Surgery and Rehabilitation*	Retrospective cohort study	United States	175	Elderly: mean 84.0 yr Nonelderly: mean 60.7 yr			Obesity, CAD, PE/DVT, COPD, asthma, stroke/TIA, atrial fibrillation, hypertension, CKD, diabetes, OSA, hypothyroidism, and anemia	Osteoarthritis and hemiathroplasty	HA, TSA, rTSA, revision
Bovonratwet^7^ 2019	Elective Total Shoulder Arthroplasty in Octogenarians: A Safe Procedure.	*Journal of the American Academy of Orthopaedic Surgeons*	Retrospective cohort study	United States	6,062 patients <70 y: 3,007; 70–79 y: 2,155; ≥80 y (octogenarians): 900	<70 yr: 61.5; 70–79 yr: 74.2; ≥80 yr: 83.1	<70 yr: 53.1%; 70–79 yr: 41.2%; ≥80 yr: 33.9%	<70 yr: 32.3; 70–79 yr: 30.9; ≥80 yr: 28.2	ASA classification	Elective primary TSA for osteoarthritis/arthrosis	Anatomic TSA and reverse TSA included
Newman^42^ 2018	Total shoulder arthroplasty in octogenarians: Is there a higher risk of adverse outcomes?	*Journal of Orthopaedics*	Retrospective cohort study	United States	10,353 patients <80 yr: 8,802; ≥80 yr: 1,551	<80 yr: mean 67 (range 19–79); ≥80 yr: mean 83 (range 80–89)	<80 yr: 45% (n = 3,998); ≥80 yr: 34% (n = 521)	<80 yr: 31.4; ≥80 yr: 28.1 (*P* < .0001)	Modified Charlson Comorbidity Index	TSA and reverse TSA	
Kim^30^ 2018	Predicting adverse events, length of stay, and discharge disposition following shoulder arthroplasty: a comparison of the Elixhauser Comorbidity Measure and Charlson Comorbidity Index.	*Seminars in Arthroplasty: JSES*	Retrospective cohort study	United States	90,491	69.2 ± 9.6 yr	41.80%	31.1 ± 6.6	Charlson Comorbidity Index (CCI) and Elixhauser Comorbidity Measure (ECM)	Elective primary TSA	Anatomic TSA and reverse TSA
Izquierdo-Fernández^27^ 2018	Reverse shoulder arthroplasty in obese patients: analysis of functionality in the medium-term.	*Archives of Orthopaedic and Trauma Surgery*	Retrospective cohort study	Spain	29 patients	78.1 yr	20.70%	31.85		Cuff-tear arthropathy and fractures/dislocations	Reverse shoulder arthroplasty
Wong^70^ 2017	The effect of patient gender on outcomes after reverse total shoulder arthroplasty.	*JSES*	Retrospective cohort study	United States	117 rTSA patients	Entire cohort: 68.7 ± 10.5 yr	48.70%	Men: 30.2 Women: 30.3 *P* = .98	Charlson Comorbidity Index (CCI) recorded Age-adjusted CCI: Men: 2.88 Women: 3.47 *P* = .027	Rotator cuff arthropathy: 44/117 (37.6%) Osteoarthritis + rotator cuff tear: 73/117 (62.4%)	Reverse total shoulder arthroplasty (rTSA) only
Ramkumar^51^ 2017	Evidence-based thresholds for the volume-value relationship in shoulder arthroplasty: outcomes and economies of scale.	*JSES*	Retrospective cohort study	United States	9,546	67 yr (±7.6 yr)	% male: 46%			Primary shoulder arthroplasty for osteoarthritis	
Issa^26^ 2016	Total Shoulder Arthroplasty Demographics, Incidence, and Complications-A National Inpatient Sample Database Study.	*Surgical Technology International*	Retrospective cohort study	United States	Total admissions: 226,227	68.9 (95% CI: 68.77-68.96)	Male: 43.6%		0: 135,918 (60.01%) 1: 63,332 (27.96%) 2: 19,171 (8.46%) ≥3: 8,074 (3.56%)		
Weinreb^69^ 2016	Shoulder Arthroplasty: Disposition and Perioperative Outcomes in Patients With and Without Rheumatoid Arthritis.	*The American Journal of Orthopedics*	Retrospective cohort study	United States	Sample size: Total: 34,970 Rheumatoid arthritis: 1,674 Non-RA: 33,296	RA: 66.39 ± 11.39 yr; Median 68 (IQR 60–75) Non-RA: 69.07 ± 9.99 yr; Median 70 (IQR 63–76) *P* < .001	RA: 24.49% Non-RA: 45.59% *P* < .001			Rheumatoid arthritis Non-RA indications not broken down	Reverse and traditional
Singh^59^ 2015	Persisting Racial Disparities in Total Shoulder Arthroplasty Utilization and Outcomes.	*Journal of Racial and Ethnic Health Disparities*	Retrospective cohort study	United States	White: 176,141 Black: 7,694 Total: 183,835	White: 69.1 yr Black: 64.2 yr (*P* < .0001)	White: 45.1% Black: 29.0% (*P* < .0001)		Deyo-Charlson index ≥2: White 8.5% vs. Black 16.7% (*P* < .0001) Underlying diagnosis: Rheumatoid arthritis: White 1.7% vs. Black 3.0% (*P* < .0001) Avascular necrosis: White 1.7% vs. Black 6.1% (*P* < .0001)	Osteoarthritis (majority, percentages not specified) Rheumatoid arthritis and avascular necrosis	
Kandil^28^ 2016	Blood transfusion after total shoulder arthroplasty: Which patients are at high risk?	*International Journal of Shoulder Surgery*	Retrospective cohort study	United States	Total: 51,191 TSA patients	68.58 ± 11.31 yr	44.4%		Charlson–Deyo score: Transfusion: 0.57 ± 1.08 No transfusion: 0.28 ± 0.67 Overall: 0.03 ± 0.70 Individual comorbidities: Obesity: 8.9% vs. 9.8% (overall 9.7%) Smoking: 4.4% vs. 6.4% (overall 6.2%) Diabetes: 18.9% vs. 14.5% (overall 15.7%) Ischemic heart disease: 21.2% vs. 14.2% (overall 14.6%) CHF: 7.7% vs. 2.5% (overall 2.9%) COPD: 18.3% vs. 15.2% (overall 15.4%) RA: 2.3% vs. 2.9% (overall 2.9%) Depression: 11.5% vs. 8.7% (overall 8.9%) Hypertension (uncomplicated): 60.1% vs. 58.9% (overall 59.0%) Hematologic risk factors: Alcohol abuse: 1.0% vs. 0.7% (overall 0.8%) Liver disease: 3.5% vs. 0.9% (overall 1.1%) Coagulation disorders: 3.5% vs. 0.9% (overall 1.1%) Blood loss anemia: 34.8% vs. 5.0% (overall 7.5%) Deficiency anemia: 29.1% vs. 6.6% (overall 8.0%) All *P* < .001 other than HTN and alcohol abuse		
Sivasundaram^62^ 2016	Preoperative risk factors for discharge to a postacute care facility after shoulder arthroplasty.	*JSES*	Retrospective cohort study	United States	Total: 103,798 patients TSA: 58,937 rTSA: 44,893	69.7 ± 9.8 yr	43.4%		Number of comorbidities: ≤3: 31.3% overall; 35.1% routine; 19.0% PAC 4–6: 44.1% overall; 43.9% routine; 42.8% PAC 7–9: 18.9% overall; 16.7% routine; 26.4% PAC ≥9: 5.8% overall; 4.3% routine; 11.9% PAC (*P* < .001) Individual comorbidities (multivariable analysis, ORs for PAC discharge): Hypertension: OR 0.95 (95% CI 0.84–1.08, *P* = .656) CHF: OR 2.00 (95% CI 1.44–2.77, *P* < .001) Diabetes: OR 1.51 (95% CI 1.29–1.77, *P* < .001) Peripheral vascular disease: OR 1.05 (95% CI 0.72–1.52, *P* = .240) CKD: OR 1.14 (95% CI 0.92–1.41, *P* = .238)	Osteoarthritis: 60.3% Rotator cuff arthropathy: 25.1% Humeral fractures: 11.7% Revision arthroplasties: 1.76% (1827 patients)	TSA: 56.8% overall; 62.9% routine; 36.6% PAC rTSA: 43.2% overall; 37.1% routine; 63.4% PAC (*P* < .001)
Basques^3^ 2015	Length of Stay and Readmission After Total Shoulder Arthroplasty: An Analysis of 1,505 Cases.	*The American Journal of Orthopedics*	Retrospective cohort study	United States	Total cohort: 1,505 TSA patients	60–69 yr: 558 (37.1%) 70–79 yr: 625 (41.5%) ≥80 yr: 322 (21.4%)	41.10%	<30: 802 (53.3%) 30–35 (class I obesity): 400 (26.6%) 35–40 (class II obesity): 182 (12.1%) ≥40 (class III obesity): 121 (8.0%)	ASA class 3–4: 802 (53.3%) Individual comorbidities: Diabetes: 266 (17.7%) Smoking: 93 (6.2%) Pulmonary disease: 176 (11.7%) Heart disease: 122 (8.1%) Hypertension: 1,092 (72.6%) Steroid use: 80 (5.3%) Dependent functional status: 48 (3.2%)	Nonfracture: 1,445 (96.0%) Fracture-associated: 60 (4.0%)	Primary TSA only + reverse TSA (but wasn't separated)
Matsen^36^ 2015	Factors Affecting Length of Stay, Readmission, and Revision After Shoulder Arthroplasty: A Population-Based Study.	*The Journal of Bone & Joint Surgery*	Retrospective cohort study	United States	Total cohort: 17,311 TSA: 8,823 HA: 7,894		35.60%		Charlson Comorbidity Index: 0: TSA 8,519; HA 7,542 1: TSA 262; HA 212 >1: TSA 42; HA 140 Specific comorbidities (TSA vs. HA): Hypertension: 5 vs. 4 Infection: 0 vs. 1 CNS: 211 vs. 294 Malignancy: 114 vs. 196 Endocrine: 1,238 vs. 1,114 Rheumatologic: 416 vs. 224 Overweight: 249 vs. 115 Substance abuse: 47 vs. 79 Pulmonary: 123 vs. 91 Cardiac: 951 vs. 802 GI: 202 vs. 152 Liver/kidney: 128 vs. 123	Fracture, inflammatory, osteoarthritis, trauma, cuff tear, osteonecrosis, miscellaneous	Primary TSA and HA
Singh^60^ 2015	Does hospital volume predict outcomes and complications after total shoulder arthroplasty in the US?	*Arthritis Care & Research*	Retrospective database study	United States	Total cohort: 256,934	<5: 68.9 yr 5–9: 69.1 10–14: 68.6 15–24: 68.8 ≥25: 68.5 *P* < .0001	<5: 40.2% 5–9: 43.3% 10–14: 43.2% 15–24: 44.8% ≥25: 45.6% *P* < .0001		Deyo-Charlson score by annual hospital TSA volume (1998–2011) Score 0: <5 TSA/yr: 64.1% 5–9 TSA/yr: 64.0% 10–14 TSA/yr: 64.1% 15–24 TSA/yr: 64.0% ≥25 TSA/yr: 63.7% *P* = .98 Score 1: <5 TSA/yr: 27.9% 5–9 TSA/yr: 27.9% 10–14 TSA/yr: 27.3% 15–24 TSA/yr: 27.6% ≥25 TSA/yr: 27.2% *P* = .81 Score ≥2: <5 TSA/yr: 8.0% 5–9 TSA/yr: 8.1% 10–14 TSA/yr: 8.6% 15–24 TSA/y: 8.5% ≥25 TSA/yr: 9.1% *P* = .11	Underlying diagnosis by annual hospital TSA volume (1998–2011) Osteoarthritis: <5 TSA/yr: 76.4% 5–9 TSA/yr: 78.3% 10–14 TSA/yr: 79.9% 15–24 TSA/yr: 81.3% ≥25 TSA/yr: 77.3% *P* = .001 Rheumatoid arthritis: <5 TSA/yr: 2.2% 5–9 TSA/yr: 2.5% 10–14 TSA/yr: 2.0% 15–24 TSA/yr: 1.6% ≥25 TSA/yr: 1.8% *P* = .007 Fracture: <5 TSA/yr: 6.3% 5–9 TSA/yr: 3.5% 10–14 TSA/yr: 2.9% 15–24 TSA/yr: 2.3% ≥25 TSA/yr: 2.1% *P* < .0001 Avascular necrosis: <5 TSA/yr: 2.7% 5–9 TSA/yr: 2.2% 10–14 TSA/yr: 1.8% 15–24 TSA/yr: 1.8% ≥25 TSA/yr: 1.5% *P* < .0001 Other diagnoses: <5 TSA/yr: 12.5% 5–9 TSA/yr: 13.6% 10–14 TSA/yr: 13.4% 15–24 TSA/yr: 13.1% ≥25 TSA/yr: 17.3% *P* < .0001	
Pappou^47^ 2014	Outcomes and Costs of Reverse Shoulder Arthroplasty in the Morbidly Obese: A Case Control Study.	*The Journal of Bone & Joint Surgery*	Retrospective cohort study	United States	Total cohort: 84 (21 morbidly obese vs. 63 matched controls) Subgroups: Morbidly obese (BMI ≥40): 21 Non-obese controls (BMI <30): 63	Morbidly obese: 69 ± 7 yr Controls: 71 ± 6 yr *P* = .152	Morbidly obese: 19% (4/21) Controls: 21% (13/63)	Morbidly obese: 43.2 ± 3.1 Controls: 27.1 ± 7.6 (*P* < .0001)	Charlson-Deyo Index (CDI): Morbidly obese: 2 ± 1 Controls: 1 ± 1 (*P* = .025) Total comorbidities (excluding obesity): Morbidly obese: 6 ± 2 Controls: 4 ± 2 (*P* = .001) Specific comorbidities: Obstructive sleep apnea (yes vs. no): 10 vs. 2 (OR 27.7; 95% CI 5.3–144.1; *P* = .0001) Recent corticosteroid use: 4 vs. 5 (OR 2.73; 95% CI 0.7–11.3; *P* = .17)	Cuff tear arthropathy (CTA): 17 vs. 51 Massive rotator cuff tear (MRCT): 2 vs. 6 Rheumatoid arthritis (RA): 2 vs. 6 *P* = 1.0 (Fisher test)	Primary reverse shoulder arthroplasty
Griffin^20^ 2014	Patient age is a factor in early outcomes after shoulder arthroplasty.	*JSES*	Retrospective database study	United States	Total cohort: 58,790 Subgroups: ≤50 yr: 4,430 51–79 yr: 43,758 ≥80 yr: 10,602	≤50 yr: 42.64 ± 7.44 51–79 yr: 67.81 ± 7.62 ≥80 yr: 83.71 ± 3.29 Overall: 68.78 ± 11.91 (*P* < .001)	≤50 yr: 58.8% 51–79 yr: 39.9% ≥80 yr: 21.9% Overall: 38.0% (*P* < .001)		Number of diagnoses: ≤50 yr: 4.31 ± 2.9 51–79 y: 5.81 ± 3.24 ≥80 yr: 6.86 ± 3.42 Overall: 5.89 ± 3.30 (*P* < .001) Number of procedures: ≤50 yr: 1.81 ± 1.49 51–79 yr: 1.63 ± 1.13 ≥80 yr: 1.78 ± 1.27 Overall: 1.67 ± 1.19 (*P* < .001) Deyo score: ≤50 yr: 0.34 ± 1.09 51–79 y: 0.32 ± 0.80 ≥80 yr: 0.40 ± 0.81 Overall: 0.34 ± 0.83 (*P* < .001) Obstructive sleep apnea: ≤50 yr: 3.7% 51–79 yr: 5.3% ≥80 yr: 1.5% Overall: 4.5% (*P* < .001)		TSA
Griffin^21^ 2014	Morbid obesity in total shoulder arthroplasty: risk, outcomes, and cost analysis.	*JSES*	Retrospective cohort study	United States	Total cohort: 31,924 Subgroups: Non-obese: 29,536 (92.5%) Obese: 1,805 (5.7%) Morbidly obese: 583 (1.8%)	Non-obese: 68.3 ± 10.9 y Obese: 66.8 ± 9.2 y Morbidly obese: 64.9 ± 9.1 y Total: 68.7 ± 10.8 y *P* < .001	Non-obese: 44.5% male Obese: 35.2% male Morbidly obese: 31.6% male Total: 43.7% male *P* < .001		Deyo score: Non-obese: 0.28 ± 0.69 Obese: 0.28 ± 0.69 Morbidly obese: 0.38 ± 0.75 Total: 0.28 ± 0.69 *P* = .004 Number of diagnoses: Non-obese: 5.31 ± 3.06 Obese: 7.78 ± 3.17 Morbidly obese: 8.26 ± 3.36 Total: 5.51 ± 3.15 *P* < .001 Number of procedures: Non-obese: 1.52 ± 0.97 Obese: 1.54 ± 0.98 Morbidly obese: 1.60 ± 1.03 Total: 1.52 ± 0.98 *P* = .086 Specific comorbidity: obstructive sleep apnea (OSA) Non-obese: 4.0% Obese: 15.6% Morbidly obese: 27.8% Total: 5.1% *P* < .001		
Khokhar^29^ 2024	Does morbid obesity negatively impact perioperative outcomes following elective reverse shoulder arthroplasty?: a propensity-matched comparative study	*Journal of Shoulder and Elbow Surgery International*	Retrospective cohort comparison	United States	Total: 175	Overall: 71 (66, 76)Morbidly obese: 68 (64, 73) Non-morbidly obese (full cohort): 71 (66.5, 77) Non-matched *P* = .12 Matched non-morbidly obese: 68 (65, 73) Matched *P*: 0.80	Morbidly obese: 16% Non-morbidly obese: 26% Matched non-morbidly obese: 10%	Morbidly obese: 42.4 (41.0, 45.1) Non-morbidly obese: 30.3 (27.2, 33.1) Matched non-morbidly obese: 30.6 (26.4, 32.7)	Overall cohort (n = 175) mFI-5 Score 0–1 BMI ≥40: 8 (42%) BMI <40: 76 (49%) *P* = .59 Score ≥2 BMI ≥40: 11 (58%) BMI <40: 80 (51%) Smoking status Never BMI ≥40: 6 (32%) BMI <40: 87 (56%) *P* = .10 Former BMI ≥40: 11 (58%) BMI <40: 53 (34%) Current BMI ≥40: 2 (10%) BMI <40: 16 (10%) Prior ipsilateral surgery None BMI ≥40: 16 (84%) BMI <40: 112 (72%) *P* = .85 Arthroscopy BMI ≥40: 3 (16%) BMI <40: 35 (22%) Open surgery BMI ≥40: 0 (0%) BMI <40: 5 (3%) Open surgery/arthroscopy BMI ≥40: 0 (0%) BMI <40: 4 (3%) Age-adjusted Charlson comorbidity index (ACCI) BMI ≥40: 4 (IQR 3–5) BMI <40: 4 (IQR 3–5) *P* = .88 Propensity matched cohort (n = 60) mFI-5 Score 0–1 BMI ≥40: 8 (42%) BMI <40: 14 (34%) *P* = .55 Score ≥2 BMI ≥40: 11 (58%) BMI <40: 27 (56%) Smoking status Never BMI ≥40: 6 (32%) BMI <40: 23 (56%) *P* = .19	RTC-deficient arthritis, GH OA, Chronic RTC tear, Other	Primary rTSA
									Former BMI ≥40: 11 (58%) BMI <40: 14 (34%) Current BMI ≥40: 2 (10%) BMI <40: 4 (10%) Prior ipsilateral surgery None BMI ≥40: 16 (84%) BMI <40: 24 (59%) *P* = .25 Arthroscopy BMI ≥40: 3 (16%) BMI <40: 12 (29%) Open surgery BMI ≥40: 0 (0%) BMI <40: 3 (7%) Open surgery/arthroscopy BMI ≥40: 0 (0%) BMI <40: 2 (5%) Age-adjusted Charlson comorbidity index (ACCI) BMI ≥40: 4 (IQR 3–5) BMI <40: 4 (IQR 3–6) *P* = .75		
Destine^16^ 2024	Effect of hospital length of stay on functional outcomes and readmission after total shoulder arthroplasty	*JSES*	Retrospective cohort study	United States	Total: 217	Overall: 67.5 ± 8.85 yr >24 h: 70.1 ± 10.4 yr <24 h: 66.7 ± 8.10 yr (*P* = .030)	Overall: 98 (45.2%) >24 h: 24 (42.9%) <24 h: 74 (46.0%) (*P* = .805)	Overall: 30.4 ± 5.00 >24 h: 31.1 ± 3.83 <24 h: 30.2 ± 5.33 (*P* = .108)	Charlson comorbidity index (CCI) - group characteristics by LOS Overall: 3.10 ± 1.64 >24 h: 4.02 ± 2.07 <24 h: 2.78 ± 1.33 (*P* = .001) Group characteristic by CCI thresholds CCI <3 - 1.62 ± 0.58 CCI ≥3 - 4.08 ± 1.36 *P* < .001 CCI <4 - 2.19 ± 0.81 CCI ≥4 - 5.04 ± 1.23 *P* < .001		rTSA and aTSA
Miltenberg^39^ 2024	Prediction of overnight stay following shoulder arthroplasty utilizing machine learning	*Journal of Shoulder and Elbow Surgery*	Retrospective cohort study	United States	Total: 5,811 TSA patients	18–60 yr: OHS 9.72% vs. SHS 15.66% 61–75 yr: OHS 51.30% vs. SHS 59.80% 76+ yr: OHS 39.98% vs. SHS 24.54% *P* < .0001 Multivariable *P* = .004	OHS: 30.67% SHS: 47.82% *P* < .0001 Same value for multivariable P	<25: OHS 21.17%, SHS 16.85% 25–30: OHS 32.61%, SHS 38.89% 31–35: OHS 21.38%, SHS 24.34% 36+: OHS 24.84%, SHS 19.92% *P* < .0001 Multivariable *P* = .012	OHS vs. SHS Anemia: 32.94% vs. 24.46%, *P* < .0001, multivariable *P* < .0001, OR 1.43 (2.21–1.69), +43% risk ASA grade 3–5: 72.25% vs. 54.86%, *P* < .0001, multivariable *P* < .0001, OR 1.88 (1.58–2.23), +88% risk CHF: 5.40% vs. 2.99%, *P* = .0002 COPD: 10.69% vs. 5.08%, *P* < .0001, multivariable *P* < .0001, OR 1.89 (1.44–2.47) Dependent functional status: 6.59% vs. 1.54%, *P* < .0001, multivariable *P* < .0001, OR 3.28 (2.24–4.81), +228% risk Diabetes mellitus: 23.11% vs. 17.48%, *P* < .0001, multivariable *P* < .0001, OR 2.85 (1.88–3.72), +185% risk Hypertension: 68.47% vs. 63.95%, *P* = .008 Smoking history: 9.29% vs. 9.09%, *P* = .848		Primary elective TSA
Guareschi^23^ 2024	Bariatric surgery is associated with increased mechanical complications and revision following total shoulder arthroplasty	*Journal of Shoulder and Elbow Surgery*	Retrospective cohort study	United States	Total: 2,620 patients Prior bariatric surgery: 1,310 No prior bariatric surgery: 1,310	Prior bariatric surgery: 66 ± 7.6 yr No prior bariatric surgery: 66 ± 7.6 yr	Prior bariatric surgery: 24% No prior bariatric surgery: 24%	Same for both groups <20: 0.3% 20–25: 0.5% 25–30: 4.7% 30–35: 24% 35–40: 28% 40–45: 26% 45–50: 11% 50–60: 5.4% >60: 0.8%	Prior bariatric surgery vs. no prior bariatric surgery CCI: 1.3 ± 1.6 vs. 0.9 ± 1.2 (*P* < .001) Active smoker: Prior bariatric surgery: 34% No prior bariatric surgery: 27% *P* value: <.001 Alcohol abuse: Prior bariatric surgery: 1.1% No prior bariatric surgery: 0.6% *P* value: .143 Hypertension requiring medication (*P* = .291) Prior bariatric surgery: 75% No prior bariatric surgery: 77% Diabetes mellitus (*P* = .010) Prior bariatric surgery: 22% No prior bariatric surgery: 18% Congestive heart failure (*P* < .001) Prior bariatric surgery: 6.6% No prior bariatric surgery: 3.4% Peripheral vascular disease (*P* = .002) Prior bariatric surgery: 4.0% No prior bariatric surgery: 1.9% Chronic kidney disease (*P* = .003) Prior bariatric surgery: 8.8% No prior bariatric surgery: 5.8% Chronic lung disease (*P* = .012) Prior bariatric surgery: 29% No prior bariatric surgery: 24% Chronic liver disease (*P* = .012) Prior bariatric surgery: 2.7% No prior bariatric surgery: 1.3% Calcium metabolism dysfunction (*P* < .001) Prior bariatric surgery: 7.8% No prior bariatric surgery: 3.9% Vitamin D deficiency (*P* < .001) Prior bariatric surgery: 7.6% No prior bariatric surgery: 3.5% Low bone mineral density (*P* = .816) Prior bariatric surgery: 2.7% No prior bariatric surgery: 2.4%		Mixed anatomic and reverse TSA
Dawes^15^ 2023	Medicaid payer status is associated with increased 90-day morbidity and resource utilization following primary shoulder arthroplasty: a propensity score-matched analysis	*Journal of Shoulder and Elbow Surgery*	Retrospective cohort	United States	Total: 165,810 161,147 non-Medicaid; 4,663 Medicaid (unmatched) Matched cohorts: 4,637 (control) 4,637 (Medicaid)	Unmatched Medicaid 55.29 ± 10.26 vs. non-Medicaid 69.81 ± 9.92 (*P* < .001). Matched Medicaid 55.44 ± 10.03 vs. non-Medicaid 56.13 ± 11.02 (*P* < .001)	Unmatched Control: 41.6% Medicaid: 46.0% Matched Control: 45.2% Medicaid: 46.0%		Comorbidities (Matched Cohorts - Medicaid vs. Control) Congestive heart failure — 3.3% vs. 4.0% (*P* = .087) Peripheral vascular disease — 1.5% vs. 1.6% (*P* = .678) COPD — 27.8% vs. 30.6% (*P* = .004) AIDS — 0.4% vs. 0.3% (*P* = .479) Coagulopathy — 2.1% vs. 2.3% (*P* = .569) Rheumatoid disease — 5.5% vs. 5.4% (*P* = .783) Chronic blood loss anemia — 0.5% vs. 0.8% (*P* = .100) Deficiency anemia — 8.3% vs. 8.9% (*P* = .299) Alcohol use disorder — 5.6% vs. 5.8% (*P* = .560) Drug use — 4.6% vs. 3.8% (*P* = .096) Depression — 20.0% vs. 21.4% (*P* = .086) Comorbidities (non-matched cohorts - Medicaid vs. Control) Congestive heart failure: Control 3.8, Medicaid 3.3, *P* = .131 Perivascular disease: Control 3.1, Medicaid 1.5, *P* < .001 COPD: Control 18.1, Medicaid 27.8, *P* < .001 AIDS: Control 0.1, Medicaid 0.4, *P* < .001 Coagulopathy: Control 1.7, Medicaid 2.1, *P* = .025 Rheumatoid disease: Control 5.5, Medicaid 5.5, *P* = .835 Chronic blood loss anemia: Control 0.7, Medicaid 0.5, *P* = .273 Deficiency anemia: Control 8.7, Medicaid 8.3, *P* = .268 Alcohol use disorders: Control 1.6, Medicaid 5.6, *P* < .001 Drug use: Control 0.8, Medicaid 4.6, *P* < .001 Depression: Control 14.8, Medicaid 20.0, *P* < .001		Unmatched cohort (*P* < .001) Total shoulder arthroplasty: Control 42.2% Medicaid 41.7% Reverse shoulder arthroplasty: Control 44.9% Medicaid 32.4% Hemiarthroplasty: Control 12.9% Medicaid 25.9% Matched cohort (*P* = .999) Total shoulder arthroplasty: Control 41.7% Medicaid 41.7% Reverse shoulder arthroplasty: Control 32.4% Medicaid 32.4% Hemiarthroplasty: Control 25.9 Medicaid 25.9%
Hartwell^25^ 2019	Total shoulder arthroplasty: Risk factors for a prolonged length of stay. A retrospective cohort study	*Current Orthopaedic Practice*	Retrospective database study	United States	Total: 14,339 TSA cases Subgroups: LOS ≤24 h: 6,507 (45.3%) LOS >24 h: 7,832 (54.7%)	Age (mean ± SD): Overall: 69.0 ± 9.7 yr LOS ≤24 h: 67.4 ± 9.7 LOS >24 h: 70.3 ± 9.7 *P* < .0001	% male: Overall: 44.0% LOS ≤24 h: 54.7% LOS >24 h: 45.3%	Overall: 31.1 ± 6.9 LOS ≤24 h: 31.0 ± 6.5 LOS >24 h: 31.1 ± 7.1	BMI: LOS ≤24 h: 31.0 ± 6.5 LOS >24 h: 31.1 ± 7.1, *P* = .29 - Diabetes: LOS ≤24 h: 233 (29.1%) LOS >24 h: 568 (70.9%), *P* < .0001 Smoking: LOS ≤24 h: 730 (48.3%) LOS >24 h: 781 (51.7%), *P* = .016 Chronic obstructive pulmonary isease (COPD): LOS ≤24 h: 299 (32.5%) LOS >24 h: 620 (67.5%), *P* < .0001 Congestive heart failure (CHF): LOS ≤24 h: 10 (14.7%) LOS >24 h: 58 (85.3%), *P* < .0001 Hypertension: LOS ≤24 h: 4,097 (42.7%) LOS >24 h: 5,489 (57.3%), *P* < .0001 - Dialysis: LOS ≤24 h: 12 (19.7%) LOS >24 h: 49 (80.3%), *P* < .0001 Steroids: LOS ≤24 h: 284 (40.3%) LOS >24 h: 421 (59.7%), *P* = .0059 Bleeding disorders: LOS ≤24 h: 127 (33.0%) LOS >24 h: 258 (67.0%), *P* < .0001 American Society of Anesthesiologists (ASA classification, mean ± SD): LOS ≤24 h: 2.4 ± 0.6 LOS >24 h: 2.6 ± 0.6, *P* < .0001		
Lyman^35^ 2005	The association between hospital volume and total shoulder arthroplasty outcomes	*Clinical Orthopaedics and Related Research*	Retrospective cohort study	United States	1,307	65.6	43.30%		Adjusted Charlson Comorbidity 0: 994 (76.1%) 1: 198 (15.1%) ≥2: 115 (8.8%) Higher Charlson associated with increased risk of readmission; *P* < .001		
Hammond^24^ 2003	Surgeon Experience and Clinical and Economic Outcomes for Shoulder Arthroplasty	*The Journal of Bone & Joint Surgery*	Retrospective cohort study	United States	1868	Mean - 68.1	31.8		CCI	Pertinent diagnoses assessed were OA, RA, and fractures of the humerus	
Lee^32^ 2020	Preoperative hyponatremia is associated with postoperative major morbidity, prolonged length of stay, non-home discharge, and readmission in total shoulder arthroplasty	*Journal of Shoulder and Elbow Surgery*	Retrospective cohort study	United States	Total sample size: 12,437 patients.	Age: Overall: 69.4 ± 9.6 yr Hyponatremia: 71.4 ± 9.6 yr Normonatremia: 69.3 ± 9.6 y *P* < .001	% male: Overall: 43.6%. Hyponatremia: 33.3%. Normonatremia: 44.4%. *P* < .001	BMI (mean ± SD): Overall: 31.2 ± 6.9. Hyponatremia: 29.6 ± 6.5. Normonatremia: 31.3 ± 6.9. *P* < .001	ASA		Anatomic TSA and reverse TSA
Vanderham^66^ 2024	Psychiatric comorbidities are an independent risk factor for perioperative and postoperative complications in patients undergoing primary total shoulder arthroplasty	*Journal of Shoulder and Elbow Surgery*	Retrospective database analysis	United States	Total sample size: 128,382 primary TSA discharges Subgroup sizes: Mental health disorder (MHD): 30,336 No MHD: 98,046	MHD: 68 ± 8.9 yr No MHD: 71 ± 8.9 yr *P* < .001 for both	MHD: 30% No MHD: 49% *P* < .001 for both		Charlson–Deyo Index		Primary TSA - both anatomic and reverse
Singh^57^ 2014	The effect of surgeon and hospital volume on shoulder arthroplasty perioperative quality metrics	*Journal of Shoulder and Elbow Surgery*	Prospective registry-based multicenter observational study	United States	Total - 1176 TSA: 711 Hemiarthroplasty: 277 Reverse TSA: 188	TSA: 69.1 ± 8.93 HA: 65.9 ± 11.84 rTSA: 74.9 ± 7.67 overall *P* < .0001	TSA: 49.1% HA: 50.9% RTSA: 42%	TSA: 30.3 ± 5.89 HA: 30.0 ± 6.17 rTSA: 28.4 ± 5.62 Overall *P* = .0003		Osteoarthritis: TSA (682) 96%, HA (215) 77.6%, rTSA (4) 2.1% Cuff tear arthropathy: TSA (0) 0%, HA (32) 11.6%, rTSA (168) 89.4% Osteonecrosis, avascular necrosis: TSA (12) 1.7%, HA (17) 6.1%, rTSA (1) 0.5% Rheumatoid arthritis: TSA (17) 2.4%, HA (7) 2.5%, rTSA (1) 0.5% Rotator cuff tear: TSA (0) 0%, HA (2) 0.7%, rTSA (12) 6.4% Instability: TSA (0) 0%, HA (4) 1.4%, rTSA (2) 1.1%	Anatomic TSA, reverse TSA, and hemiarthroplasty
Koh^31^ 2018	Thirty-day Complications and Readmission Rates in Elderly Patients After Shoulder Arthroplasty	*JAAOS: Global Research and Reviews*	Retrospective cohort study	United States	Total - 11,450 Hemiarthroplasty - 1956 (17.1%) Total or reverse shoulder arthroplasty - 9,494 (82.9%)	≥80 yr ("elderly"): 1,708 (15.0%) 65–79 yr ("older"): 6,073 (53.0%) <65 yr ("younger"): 3,669 (32.0%)- link broken so I can't get the mean, SD, median (IQR)	43.60%	*P* value: <0.001 Normal (18.5–24 kg/m^2^) ≥80 yr: 508 (13.9%) 65–79 yr: 930 (15.4%) <65 yr: 431 (25.4%) Underweight (<18.5 kg/m^2^) ≥80 yr: 27 (0.7%) 65–79 yr: 40 (0.7%) <65 yr: 28 (1.6%) Overweight (25–29 kg/m^2^) ≥80 yr: 1,053 (28.8%) 65–79 yr: 1,919 (31.7%) <65 yr: 700 (41.2%) Obese (≥30 kg/m^2^) ≥80 yr: 2,070 (56.6%) 65–79 yr: 3,164 (52.3%) <65 yr: 539 (31.7%)	ASA class (1–2 vs. ≥ 3) - hypertension, diabetes, pulmonary disease, cardiac disease (CHF or MI history), tobacco use, chronic steroid use, pre-operative functional status (independent vs. nonindependent)	Osteoarthritis <65: 2,342 (63.8%) 65–79: 4,226 (69.6%) ≥80: 1,121 (65.6%) Avascular necrosis <65: 173 (4.7%) 65–79: 68 (1.1%) ≥80: 8 (0.5%) Cuff arthropathy <65: 240 (6.5%) 65–79: 536 (8.8%) ≥80: 162 (9.5%) Revision arthroplasty <65: 166 (4.5%) 65–79: 191 (3.1%) ≥80: 46 (2.7%) Fracture <65: 258 (7.0%) 65–79: 454 (7.5%) ≥80: 232 (13.6%) Unknown <65: 490 (13.4%) 65–79: 598 (9.8%) ≥80: 139 (8.1%)	Hemiarthroplasty <65: 890 (24.3%) 65–79: 798 (13.1%) ≥80: 268 (15.7%) Shoulder arthroplasty (TSA/rTSA) <65: 2,779 (75.7%) 65–79: 5,275 (86.9%) ≥80: 1,440 (84.3%)
Singh^61^ 2015	Age-related differences in the use of total shoulder arthroplasty over time USE AND OUTCOMES	*Bone & Joint Journal*	Retrospective cohort study using administrative database - National Inpatient Sample (NIS), 1998–2010	United States	46,413				Deyo-Charlson Comorbidity Index		Primary TSA
Schairer^54^ 2015	National utilization of reverse total shoulder arthroplasty in the United States	*Journal of Shoulder and Elbow Surgery*	Retrospective national database analysis	United States	Total weighted estimate - 66,485 rTSA - 21,692 TSA - 29,359 Hemiarthroplasty - 15,434	rTSA: 72.7 ± 16.1 TSA: 67.4 ± 15.1 hemi: 66.8 ± 28.1	rTSA: 36.4%; TSA: 49.6%; Hemi: 37.0%		Number of comorbidities - mean: rTSA: 2.18; TSA: 1.86; hemi: 2.21	Osteoarthritis 93.4% (n = 27,420) Avascular necrosis 2.0% (n = 594) Inflammatory arthritis 1.2% (n = 356) Proximal humerus fracture 1.2% (n = 349) Rotator cuff tear 1.0% (n = 306) Revision arthroplasty 0.5% (n = 155) Malunion/nonunion 0.4% (n = 116) Instability 0.1% (n = 41) Malignancy 0.0% (n = 14) Osteomyelitis 0.0% (n = 5)	Anatomic TSA
Yin^71^ 2019	The Effect of Race on Early Perioperative Outcomes After Shoulder Arthroplasty: A Propensity Score Matched Analysis	*Orthopedics*	Retrospective cohort study	United States	2,785	63.9 ± 11.7 yr	39.00%	Body mass index .977 Normal (18.5-24 kg/m2) 333 (14.9) 90 (16.2) Underweight (<18.5 kg/m2) 21 (0.9) 4 (0.7) Overweight (25-29 kg/m2) 545 (24.5) 136 (24.4) Obese class I (30-34 kg/m2) 505 (22.7) 126 (22.6) Obese class II (35-40 kg/m2) 374 (16.8) 90 (16.2) Obese class III (40+ kg/m2) 450 (20.2) 111 (19.9)	ASA classification - 1–2: 32.5%, ≥3: ∼67.5%; no other comorbidity index used	OA (61.5%), AVN (6.1–8.1%), cuff arthropathy (11%), revision, trauma, unknown	Both reverse and anatomic performed - but reverse vs. anatomic not separated
Sharma^56^ 2024	Social determinants of health disparities impact postoperative complications in patients undergoing total shoulder arthroplasty	*JSES*	Retrospective case-control design	United States	Total n = 16,046 After matching: 8,023 with SDHD vs. 8,023 without SDHD (controls)	SDHD: 67.18 ± 8.58 Control: 67.18 ± 8.49	SDHD: 31% Control: 31%		Charlson Comorbidity Index (CCI) SDHD: 2.27 ± 2.30 Control: 2.27 ± 2.30 *P* > .999 Hypertension SDHD: 7,353 Control: 7,353 *P* > .999 Ischemic heart disease (IHD) SDHD: 2,380 Control: 2,380 *P* > .999 Chronic obstructive pulmonary disease (COPD) SDHD: 3,787 Control: 3,787 *P* > .999 Diabetes mellitus SDHD: 4,281 Control: 4,281 *P* > .999 Chronic kidney disease (CKD) SDHD: 2,174 Control: 2,174 *P* > .999 Rheumatoid arthritis (RA) SDHD: 638 Control: 638 *P* > .999 Tobacco use SDHD: 3,952 Control: 3,952 *P* > .999 Obese SDHD: 2,574 Control: 2,574 *P* > .999 Morbidly obese SDHD: 1853 Control: 1853 *P* > .999		Primary total shoulder arthroplasty

*HA*, hemiarthroplasty; *BMI*, body mass index; *TSA*, total shoulder arthroplasty; *OA*, osteoarthritis; *LOS*, length of stay; *SDHD*, social determinants of health disparities; *OARA*, Outpatient Arthroplasty Risk Assessment; *CKD*, chronic kidney disease; *PE*, pulmonary embolism; *DVT*, deep vein thrombosis; *SOT*, solid organ transplant; *IP*, inpatient; *IA*, inflammatory arthritis; *HTN*, hypertension; *GH*, glenohumeral; *RTC*, rotator cuff; *HbA1c*, hemoglobin A1c; *GI*, gastrointestinal; *mFI*, modified frailty index; *PAC*, post-acute care (facility); *TIA*, transient ischemic attack; *SD*, standard deviation; *IQR*, interquartile range; *CI*, confidence interval; *OR*, odds ratio; *IRR*, incidence rate ratio.

**Table II:** Study summary.

Author (yr)	Title	Data source	Aims/methods	Conclusion
Nazzal^41^ 2025	Predictors of failing same-day discharge after shoulder arthroplasty: developing a model to improve outcomes and reduce health care cost.	Single-center institutional retrospective review of consecutive single-surgeon shoulder arthroplasty cases, Jan 2019–Jun 2023	Retrospective cohort study of consecutive shoulder arthroplasty cases intended for same-day discharge, evaluating demographic, clinical, and perioperative factors associated with failure of same-day discharge through multivariable logistic regression to develop a predictive model for post-operative hospital admission.	Older age, rotator cuff arthropathy, female sex, pre-operative opioid use, and prolonged anesthesia duration significantly increased the likelihood of failing same-day discharge. These findings support risk-stratified selection of candidates for outpatient arthroplasty and highlight the importance of coordinated perioperative planning to optimize resource utilization.
Areti^2^ 2025	Impact of morbid obesity on postoperative outcomes in reverse total shoulder arthroplasty: A national inpatient sample analysis.	National Inpatient Sample (NIS), 2016–2019	Retrospective cohort analysis of a national inpatient database evaluating whether morbid obesity is associated with adverse perioperative outcomes following reverse total shoulder arthroplasty, using comparative statistics and multivariate logistic regression to assess differences in complications, discharge status, and length of stay.	Morbid obesity was associated with higher complication rates, prolonged hospitalization, and increased likelihood of non-routine discharge after reverse total shoulder arthroplasty, highlighting the need for tailored perioperative optimization and post-operative management in this high-risk population.
Burns^9^ 2024	Prior nonarthroplasty shoulder surgery and modifiable risk factors negatively affect patient outcomes after shoulder arthroplasty.	Single-institution, single-surgeon retrospective cohort, Jan 2015–Dec 2019	Retrospective single-surgeon cohort study evaluating whether a history of nonarthroplasty shoulder surgery influences post-operative complications, costs, and patient-reported outcomes after primary shoulder arthroplasty, with multivariable analysis examining the effect of modifiable risk factors on clinical results.	Patients with prior shoulder surgery experienced inferior subjective outcomes and a higher likelihood of perioperative complications. Modifiable risk factors, particularly tobacco and opioid use, further worsened patient-reported scores, underscoring the value of targeted pre-operative optimization in this population.
Gammel^18^ 2025	Medicaid status is independently predictive of increased complications, readmission, and mortality following primary total shoulder arthroplasty.	Nationwide Readmissions Database (NRD), 2016–2020	Retrospective national claims database study assessing whether Medicaid insurance independently predicts post-operative complications, readmission, revision, mortality, and resource utilization after elective primary total shoulder arthroplasty, using propensity score matching and multivariable logistic regression to control for baseline disparities.	Medicaid status independently increased the risk of post-operative complications, readmission, and mortality, and was associated with greater hospital resource use. Insurance-based disparities should inform perioperative planning and counseling for patients undergoing primary total shoulder arthroplasty.
Sohn^63^ 2024	The Influence of Solid Organ Transplant on Inpatient Complications, Length of Stay, and Hospital Costs in Reverse Shoulder Arthroplasty Patients.	National Inpatient Sample (NIS), HCUP, Jan 2016–Dec 2019	Retrospective national inpatient database analysis comparing demographics, complication rates, length of stay, and hospital costs in solid organ transplant recipients undergoing reverse shoulder arthroplasty, using propensity matching to account for baseline comorbidities.	Solid organ transplant recipients undergoing reverse shoulder arthroplasty had similar overall complication rates but demonstrated a markedly increased risk of acute renal failure, along with longer hospitalizations and higher inpatient costs, emphasizing the need for tailored perioperative management in this population.
Raji^50^ 2024	Same-day discharge vs. inpatient total shoulder arthroplasty: an age stratified comparison of postoperative outcomes and hospital charges.	Single-institution retrospective case-control cohort, single surgeon, at a community hospital within a tertiary referral health-care system, Jan 2018–Oct 2021	Retrospective comparison of same-day discharge versus inpatient primary total shoulder arthroplasty, with age-stratified analysis of 90-d complications, emergency utilization, readmissions, reoperations, hospital charges, and length of stay to evaluate safety and efficiency, particularly in super-elderly patients.	Same-day discharge yielded shorter length of stay without increasing 90-d complications, readmissions, or costs, including among older and super-elderly patients, supporting outpatient total shoulder arthroplasty as a safe and efficient option when appropriately selected.
Mayfield^37^ 2024	Shoulder arthroplasty for inflammatory arthritis is associated with higher rates of medical and surgical complications: a nationwide matched cohort analysis from 2016-2020.	Premier Healthcare Database (PHD), Jan 2016–Dec 2020	Retrospective analysis of a national hospital database comparing outcomes of anatomic and reverse total shoulder arthroplasty in patients with inflammatory arthritis versus matched osteoarthritis controls, using 1:8 matched cohorts and multivariable regression to evaluate 90-d surgical and medical complications	Inflammatory arthritis was associated with a distinct and higher-risk complication profile, including increased infection, implant-related mechanical issues, thromboembolic events, and anemia, supporting multidisciplinary optimization and tailored perioperative management for these patients undergoing shoulder arthroplasty.
Gruson^22^ 2023	Does Morbid Obesity (BMI â‰¥40 kg/m2) Impact Operative Time, Blood Loss, Length of Stay, or Complications Following Anatomic Total Shoulder Arthroplasty?	Retrospective review of consecutive anatomic TSA cases by a single fellowship-trained shoulder surgeon; institutional cohort, Jan 2016-March 2020 and Jan 2021-Jan 2022	Retrospective review of consecutive anatomic total shoulder arthroplasty cases assessing the impact of obesity classification on operative time, blood loss, perioperative complications, and hospital length of stay, using correlation analyses and regression modeling to identify predictors of prolonged hospitalization.	Morbid obesity did not increase operative time, blood loss, or complication rates but independently predicted longer length of stay, indicating that elevated BMI primarily affects post-operative resource utilization rather than surgical complexity in anatomic shoulder arthroplasty.
Puzzitiello^49^ 2022	Patients with limited health literacy have worse preoperative function and pain control and experience prolonged hospitalizations following shoulder arthroplasty.	Prospectively maintained single-surgeon electronic registry (OBERD, Columbia, MO, USA), urban orthopedic specialty hospital, Jan 2018–May 2021	Retrospective single-surgeon cohort study evaluating whether limited health literacy, identified pre-operatively using a validated screening tool, influences pre-operative symptom burden and perioperative outcomes after elective shoulder arthroplasty, with multivariable regression used to assess its effect on length of stay.	Limited health literacy was common and associated with worse pre-operative symptom severity and independently predicted longer hospitalization, indicating its relevance in perioperative planning and post-operative disposition after shoulder arthroplasty
Burns^8^ 2022	Modifiable risk factors increase length of stay and 90-day cost of care after shoulder arthroplasty.	Epic EMR, regional 7-hospital system, Oct 2015–Dec 2019	Retrospective review across a regional health system assessing the impact of predefined modifiable risk factors—including anemia, malnutrition, obesity, uncontrolled diabetes, tobacco use, and opioid use—on 90-d encounter charges, hospital length of stay, emergency department utilization, and readmissions following shoulder arthroplasty, using multivariable modeling to evaluate cost and resource use.	Malnutrition, obesity, and anemia significantly increased post-operative costs and resource utilization, particularly length of stay and readmissions, supporting targeted pre-operative optimization programs to reduce financial burden and improve outcomes in shoulder arthroplasty.
Lunati^34^ 2021	Preoperative depression is a risk factor for complication and increased health care utilization following total shoulder arthroplasty.	Truven MarketScan Commercial Claims and Encounters–Medicare Supplemental database, 2009–2017	Retrospective administrative database analysis comparing outcomes of primary total shoulder arthroplasty in patients with and without pre-operative depression, using multivariate adjustment to evaluate post-operative complications, prosthetic failures, and healthcare utilization within one yr.	Pre-operative depression was common and independently associated with higher complication rates, increased prosthetic and wound-related failures, and greater healthcare utilization, indicating that mental health status may influence post-operative risk and warrants consideration during pre-operative planning.
Polisetty^48^ 2021	Determining the validity of the Outpatient Arthroplasty Risk Assessment (OARA) tool for identifying patients for safe same-day discharge after primary shoulder arthroplasty.	Single-surgeon shoulder and elbow repository/medical records, Apr 2018–Oct 2019	Retrospective single-surgeon study evaluating the utility of the Outpatient Arthroplasty Risk Assessment (OARA) score for determining appropriateness for same-day discharge after primary shoulder arthroplasty, comparing score thresholds and predictive performance against ASA-PS classification and prior lower-extremity arthroplasty criteria	An OARA cutoff of ≤110 points effectively and conservatively identified candidates for same-day discharge, with low emergency utilization and acceptable readmission rates, supporting its use as a screening tool for outpatient shoulder arthroplasty.
Dacombe^13^ 2020	Predictors of length of stay following shoulder arthroplasty in a high-volume UK centre.	Single-center retrospective review; case notes, electronic patient records, and pathology systems; high-volume UK center, Jan 2012-Jan 2018	Retrospective review of shoulder arthroplasty cases at a high-volume UK center evaluating patient, comorbidity, and perioperative factors associated with inpatient length of stay, with multivariable linear regression used to identify independent predictors of prolonged hospitalization.	Advanced age, female sex, cardiac and renal comorbidities, intraoperative complications, and transfusion independently increased length of stay, supporting targeted optimization and reduction of perioperative complications to improve inpatient efficiency following shoulder arthroplasty.
Menendez^38^ 2019	Delayed hospital discharge after total shoulder arthroplasty: why, and who is at risk?	Prospectively collected institutional registry + manual medical record review; urban orthopedic specialty hospital, single fellowship-trained shoulder surgeon, Jan 2016–Nov 2017	Retrospective review of elective primary total shoulder arthroplasty assessing reasons for extended hospitalization and identifying pre-operative factors associated with prolonged length of stay, using manual chart review to classify causes and multivariable logistic regression to determine independent predictors	Most extended hospitalizations were due to pain, minor medical issues, or limited social support rather than surgical complications, with sociodemographic and psychological factors emerging as key predictors, supporting comprehensive perioperative management beyond technical care alone.
Biron^5^ 2020	A Novel Machine Learning Model Developed to Assist in Patient Selection for Outpatient Total Shoulder Arthroplasty.	American College of Surgeons National Surgical Quality Improvement Program (ACS-NSQIP), 2011–2016	Retrospective analysis of a national surgical database using a random forest machine learning model to predict short-stay outpatient candidacy after elective total shoulder arthroplasty, followed by multivariable logistic regression to identify demographic and comorbidity factors associated with length of stay.	Machine learning accurately identified patients likely to achieve short stays, with younger age and male sex favoring outpatient eligibility, while cardiopulmonary comorbidities and higher ASA classification predicted longer hospitalization, supporting its potential role in outpatient candidate selection.
Singh^58^ 2020	Insurance Payer Type and Patient Income Are Associated with Outcomes after Total Shoulder Arthroplasty.	US National Inpatient Sample (NIS), 1998–2014	Retrospective analysis of the US National Inpatient Sample evaluating whether insurance type and neighborhood-level income independently influence inpatient complications, discharge disposition, length of stay, and hospital charges after total shoulder arthroplasty, using multivariable logistic regression.	Government insurance and lower income were associated with greater resource utilization and select complications after shoulder arthroplasty, indicating that socioeconomic status and insurance type may influence post-operative risk and warrant consideration for patient counseling and perioperative planning.
Navarro^40^ 2019	Short-term outcomes after anatomic total shoulder arthroplasty in patients with osteoarthritis versus osteonecrosis.	American College of Surgeons National Surgical Quality Improvement Program (ACS-NSQIP), 2008–2015	Retrospective national surgical database study comparing short-term perioperative outcomes of anatomic total shoulder arthroplasty for osteonecrosis versus osteoarthritis, using propensity score matching and multivariable analysis to assess 30-d complications and hospital disposition.	Despite higher comorbidity burden among osteonecrosis patients, short-term perioperative outcomes were similar to osteoarthritis, suggesting no clinically meaningful differences in early safety profiles between these indications for anatomic total shoulder arthroplasty
Sabesan^53^ 2020	Relationship between hospital size and teaching status on outcomes for reverse shoulder arthroplasty.	National Inpatient Sample (NIS), ICD-9 rTSA discharges, Jan 2011-Dec 2012	National Inpatient Sample analysis of reverse shoulder arthroplasty evaluating the effect of hospital size and setting on post-operative outcomes. ICD-9 codes were used to identify cases, and length of stay, hospital charges, discharge disposition, and inpatient complications were compared. Odds ratios quantified risk of adverse events between large versus small/medium and teaching versus non-teaching hospitals.	Large hospitals were associated with greater costs, longer inpatient stay, and increased nonroutine discharge, with higher genitourinary and CNS complication rates. Non-teaching centers demonstrated lower infection rates but greater risk of post-operative anemia. As rTSA utilization expands, attention to surgical setting and hospital resource optimization remains critical to improving outcomes.
Crook^12^ 2018	Comparison of 90-Day Perioperative Outcomes in Shoulder Arthroplasty Between the Elderly and Nonelderly Patients.	Single-institution retrospective cohort; Virginia Commonwealth University; single surgeon; 2007–2015	To determine whether age ≥80 yr is an independent risk factor for post-operative healthcare utilization after shoulder arthroplasty by comparing discharge disposition, length of stay, perioperative complications, readmission, and mortality between elderly (≥80 yr) and non-elderly (<80 yr) patients.	Patients aged ≥80 yr undergoing shoulder arthroplasty experience significantly longer hospital stays and are far more likely to require discharge to a skilled nursing facility. However, advanced age alone is not associated with increased 90-d complications, readmission, or mortality, supporting shoulder arthroplasty as a safe procedure in appropriately selected elderly patients.
Bovonratwet^7^ 2019	Elective Total Shoulder Arthroplasty in Octogenarians: A Safe Procedure.	American College of Surgeons National Surgical Quality Improvement Program (ACS-NSQIP), Jan 2005–Dec 2015	To compare perioperative complications, length of stay, and 30-d readmissions in octogenarians (≥80 yr) undergoing primary total shoulder arthroplasty (TSA) with outcomes in patients aged <70 and 70–79 Patients who underwent primary TSA between 2005 and 2015 were identified in the NSQIP database and stratified into 3 age cohorts: <70, 70–79, and ≥80 y. Patient characteristics and comorbidities were compared, followed by propensity score matching. Outcomes included length of hospital stay, 30-d complications, readmissions, and reasons for readmission.	Primary TSA is safe in octogenarians, demonstrating complication rates comparable to patients aged 70–79. However, octogenarians had higher rates of readmission (particularly due to pneumonia and urinary tract infection) and slightly longer hospitalization compared with younger patients. These findings support TSA use in this population while emphasizing the need for focused pre-operative optimization and enhanced post-discharge care for patients ≥80 yr old.
Newman^42^ 2018	Total shoulder arthroplasty in octogenarians: Is there a higher risk of adverse outcomes?	American College of Surgeons National Surgical Quality Improvement Program (ACS-NSQIP)	Assessed octogenarians undergoing total shoulder arthroplasty using a national surgical quality database to compare perioperative characteristics and evaluate 30-d post-operative complications relative to younger	Octogenarians demonstrated higher odds of overall, major, and minor early complications, indicating that focused perioperative optimization strategies are essential when performing elective total shoulder arthroplasty in this population.
Kim^30^ 2018	Predicting adverse events, length of stay, and discharge disposition following shoulder arthroplasty: a comparison of the Elixhauser Comorbidity Measure and Charlson Comorbidity Index.	National Inpatient Sample (NIS), 2002–2014	Queried a national inpatient database to evaluate whether the Elixhauser Comorbidity Measure provides superior predictive accuracy to the Charlson Comorbidity Index for forecasting adverse events, resource utilization, and discharge disposition following total shoulder and reverse total shoulder arthroplasty using logistic regression models and concordance statistics.	A model incorporating the Elixhauser Comorbidity Measure demonstrated superior discrimination for mortality, complications, extended hospitalization, and nonroutine discharge, indicating its value as a more reliable tool for guiding perioperative care pathways and supporting value-based reimbursement strategies in shoulder arthroplasty.
Izquierdo-Fernández^27^ 2018	Reverse shoulder arthroplasty in obese patients: analysis of functionality in the medium-term.	Single-hospital prospective database; University Hospital Reina Sofía, Córdoba, Spain; 2012	Evaluated functional and radiographic outcomes of reverse shoulder arthroplasty in patients stratified by body mass index, comparing 2 BMI-based subgroup analyses and assessing complications, length of stay, radiographic changes, and standardized functional scores.	Patients with BMI ≥35 demonstrated significantly poorer functional results, while complication rates and radiographic findings were similar across BMI groups, indicating that severe obesity adversely affects post-operative function without increasing measurable implant-related complications.
Wong^70^ 2017	The effect of patient gender on outcomes after reverse total shoulder arthroplasty.	Single-institution prospectively collected shoulder arthroplasty outcomes database carried out by 3 sports medicine and shoulder fellowship-trained orthopedic surgeons, 2009–2015	Prospectively evaluated whether gender influences pre-operative disability and post-operative patient-reported outcomes following reverse total shoulder arthroplasty, analyzing pain, range of motion, and standardized functional and health status scores using multivariate mixed-effects regression.	Post-operative pain relief and range-of-motion improvements were comparable between genders; however, men achieved higher functional and physical health scores, suggesting that women may experience relatively greater functional limitations despite similar surgical benefits.
Ramkumar^51^ 2017	Evidence-based thresholds for the volume-value relationship in shoulder arthroplasty: outcomes and economies of scale.	New York State Statewide Planning and Research Cooperative System (SPARCS) administrative database across 157 hospitals	Applied stratum-specific likelihood ratio analysis of ROC curves to derive evidence-based surgeon and hospital volume thresholds associated with increased length of stay and cost in primary shoulder arthroplasty. Four models were constructed to identify volume strata that best discriminated risk of adverse value-related outcomes.	Identified clear low-, medium-, and high-volume categories for surgeons and hospitals, demonstrating a consistent inverse relationship between volume and both length of stay and cost. Findings support meaningful, evidence-based thresholds that define volume–value relationships in shoulder arthroplasty and inform optimization of care delivery.
Issa^26^ 2016	Total Shoulder Arthroplasty Demographics, Incidence, and Complications-A National Inpatient Sample Database Study.	National Inpatient Sample (NIS)/HCUP	Evaluated contemporary national trends in total shoulder arthroplasty using the National Inpatient Sample (1998–2010), assessing temporal changes in utilization, demographic patterns, and inpatient outcomes. Multivariable regression was applied to determine independent associations between patient factors, complications, and length of stay.	Incidence of total shoulder arthroplasty increased substantially over the study period, while complication rates remained stable and length of stay declined. Older age, female sex, and greater comorbidity burden were independently associated with higher complication risk, and several demographic and insurance factors contributed to prolonged hospitalization.
Weinreb^69^ 2016	Shoulder Arthroplasty: Disposition and Perioperative Outcomes in Patients With and Without Rheumatoid Arthritis.	National Inpatient Sample (NIS)/HCUP	Retrospective analysis of a national inpatient database comparing perioperative complication profiles and hospital disposition between patients undergoing shoulder arthroplasty with and without rheumatoid arthritis, using multivariable regression to adjust for demographic and hospital factors.	Rheumatoid arthritis was associated with increased transfusion requirements and modest differences in perioperative resource use and discharge patterns, while overall complication rates remained comparable. Further work is needed to clarify drivers of these disparities and optimize perioperative management.
Singh^59^ 2015	Persisting Racial Disparities in Total Shoulder Arthroplasty Utilization and Outcomes.	National Inpatient Sample (NIS)/HCUP, 1998–2011	Used a national inpatient database (1998–2011) to evaluate temporal trends in racial disparities in TSA utilization and post-operative outcomes, applying chi-square testing, Cochran–Armitage trend analyses, and logistic regression to compare patterns over time.	Racial disparities in TSA utilization widened over the study period, and select outcome differences persisted without evidence of improvement. Findings highlight the need for targeted strategies to reduce inequities in access and care.
Kandil^28^ 2016	Blood transfusion after total shoulder arthroplasty: Which patients are at high risk?	National Inpatient Sample (NIS)/HCUP	Used a national inpatient database to evaluate contemporary transfusion incidence in TSA and to identify pre-operative variables independently associated with transfusion. Assessed relationships between transfusion status and hospital utilization metrics.	Several pre-operative comorbidities were independently associated with transfusion, allowing clearer stratification of higher-risk patients. Transfusion was also linked to greater resource use, underscoring the importance of targeted risk counseling and perioperative optimization.
Sivasundaram^62^ 2016	Preoperative risk factors for discharge to a postacute care facility after shoulder arthroplasty.	National Inpatient Sample (NIS)/HCUP	Using a national inpatient database, the study evaluated patient and hospital characteristics associated with discharge to postacute care following total or reverse shoulder arthroplasty. Multivariate modeling was applied to identify independent predictors while adjusting for relevant covariates.	The analysis identified several demographic, clinical, and procedural factors that independently increase the likelihood of postacute care discharge, providing a basis for pre-operative risk stratification and more efficient discharge planning.
Basques^3^ 2015	Length of Stay and Readmission After Total Shoulder Arthroplasty: An Analysis of 1,505 Cases.	Single-institution retrospective review; Rush University Medical Center (1,505 cases)	The study examined patient-related predictors of extended hospital stay and 30-d readmission following primary total shoulder arthroplasty using a national surgical quality database. Bivariate and multivariate regression models were applied to evaluate associations between pre-operative characteristics and post-operative utilization outcomes.	Specific comorbidities were independently linked to prolonged hospitalization and early readmission, highlighting the value of targeted risk stratification to guide pre-operative counseling and post-operative planning.
Matsen^36^ 2015	Factors Affecting Length of Stay, Readmission, and Revision After Shoulder Arthroplasty: A Population-Based Study.	Washington State Comprehensive Hospital Abstract Reporting System (CHARS) database	Analyzed statewide administrative data from primary shoulder arthroplasty procedures to identify patient, diagnosis, and provider factors independently associated with prolonged hospitalization, 90-d readmission, and revision surgery. Multivariable models were used to determine which characteristics predicted each adverse outcome.	Distinct clinical and demographic variables were linked to extended stay, early readmission, and need for revision. Readily identifiable patient and provider characteristics may inform perioperative optimization strategies and support targeted efforts to reduce complications and associated healthcare costs.
Singh^60^ 2015	Does hospital volume predict outcomes and complications after total shoulder arthroplasty in the US?	National Inpatient Sample (NIS)/HCUP	Retrospective analysis of a national inpatient database to evaluate the association between hospital annual TSA volume and key post-operative outcomes, using predefined volume thresholds to compare discharge disposition, length of stay, and complication rates across institutions.	Higher hospital TSA volume correlated with more favorable post-operative outcomes, including reduced facility-based discharge, shorter hospitalization, and lower complication risks. Findings support volume-related quality advantages that may inform clinical decision-making and health-system planning.
Pappou^47^ 2014	Outcomes and Costs of Reverse Shoulder Arthroplasty in the Morbidly Obese: A Case Control Study.	Single-institution case-control study	The study compared post-operative outcomes, complications, discharge disposition, and hospital costs between morbidly obese patients and matched nonobese controls undergoing primary reverse shoulder arthroplasty. A joint registry cohort with minimum 2-yr follow-up was analyzed using standardized functional measures, comorbidity indices, perioperative metrics, and recorded post-operative events.	Reverse shoulder arthroplasty demonstrated comparable functional gains and complication rates in morbidly obese patients relative to nonobese controls, though obesity was associated with increased perioperative resource utilization, higher costs, and a greater likelihood of requiring postacute rehabilitation rather than home discharge.
Griffin^20^ 2014	Patient age is a factor in early outcomes after shoulder arthroplasty.	National Inpatient Sample (NIS)/HCUP, 2000–2008	Leveraged a national inpatient database to assess whether age independently influences in-hospital complications, mortality, length of stay, or hospital charges following shoulder arthroplasty, using multivariate logistic regression for risk adjustment.	Advanced age was associated with prolonged hospitalization, higher perioperative anemia rates, and modestly increased charges, while risks of major post-operative complications remained comparable across age groups. Further work is needed to clarify mechanisms linking age to early post-operative outcomes.
Griffin^21^ 2014	Morbid obesity in total shoulder arthroplasty: risk, outcomes, and cost analysis.	National Inpatient Sample (NIS)/HCUP, 1998–2008	Retrospective analysis of a national inpatient database comparing post-operative complications, mortality, and resource utilization between morbidly obese and non-obese patients undergoing total shoulder arthroplasty using multivariate logistic regression modeling.	Morbid obesity was associated with greater post-operative respiratory complications, longer hospitalization, and higher inpatient costs, while most other complication rates and early mortality showed no significant increase. Further investigation is warranted to refine risk stratification and support pre-operative counseling.
Khokhar^29^ 2024	Does computed tomography-based local soft tissue composition more accurately predict outcomes following anatomic total shoulder arthroplasty compared with body mass index?: a pilot study	Single-institution retrospective review; Montefiore Medical Center/Albert Einstein College of Medicine, Bronx, NY (consecutive primary stemless aTSA cases)	A retrospective single-center review of consecutive primary stemless aTSA cases was conducted to evaluate whether CT-derived measures of local adipose and muscle composition better predict perioperative outcomes than BMI. Standardized axial measurements of adipose, muscle, and bone area were obtained, and regression analyses examined associations between adipose-to-muscle and soft tissue-to-bone ratios with post-operative clinical metrics.	CT-based adipose distribution, particularly the adipose-to-muscle ratio, demonstrated stronger associations with perioperative outcomes than BMI alone, indicating that local soft tissue composition may serve as a more clinically informative predictor in primary aTSA.
Destine^16^ 2024	Effect of hospital length of stay on functional outcomes and readmission after total shoulder arthroplasty	Single-institution retrospective review; Thomas Jefferson University Hospital (2010–2019)	A retrospective cohort study evaluated whether post-operative length of stay (≤24 vs. > 24 h) was associated with readmissions, revisions, complications, or functional outcomes following primary total shoulder arthroplasty. Demographic, perioperative, comorbidity, and discharge variables were analyzed to identify factors predictive of prolonged hospitalization.	Prolonged post-operative stay was associated with higher comorbidity burden and increased likelihood of discharge to a non-home setting but did not influence complication rates, readmissions, revisions, or patient-reported outcomes.
Miltenberg^39^ 2024	Prediction of overnight stay following shoulder arthroplasty utilizing machine learning	ACS-NSQIP (American College of Surgeons National Surgical Quality Improvement Program)	This study evaluated multiple machine learning models to predict likelihood of overnight hospital stay after primary elective total shoulder arthroplasty using a national surgical outcomes database. Models were trained and validated against post-operative disposition categories, with performance compared using discrimination, calibration, and decision curve analyses.	Machine learning—particularly artificial neural networks—provided superior predictive accuracy over traditional regression approaches for forecasting need for post-operative admission. These models can support more precise outpatient selection, inform pre-operative counseling, and facilitate resource optimization.
Guareschi^23^ 2024	Bariatric surgery is associated with increased mechanical complications and revision following total shoulder arthroplasty	Nationwide Readmissions Database (NRD)/HCUP, 2015–2020	This study used a nationwide administrative database to compare post-operative outcomes in patients undergoing primary anatomic or reverse total shoulder arthroplasty, stratified by prior bariatric surgery. Matched cohorts were evaluated for pre-operative comorbidity profiles, perioperative resource utilization, and 180-d complication rates using multivariate adjustment to isolate the association with bariatric surgery history.	Prior bariatric surgery was associated with greater baseline metabolic derangements and substantially higher odds of early post-operative complications, revision, and implant-related failure after shoulder arthroplasty, as well as increased costs and greater need for post-operative support services.
Dawes^15^ 2023	Medicaid payer status is associated with increased 90-day morbidity and resource utilization following primary shoulder arthroplasty: a propensity score-matched analysis	National Inpatient Sample (NIS) and Nationwide Readmissions Database (NRD)/HCUP, 2011–2019	This study evaluated whether Medicaid payer status independently influences 90-d readmission, reoperation, hospital length of stay, and direct costs after primary shoulder arthroplasty. Using the National Readmission Database, patients undergoing aTSA, rTSA, or HA were identified and matched 1:1 by propensity scores to control for baseline differences. Outcomes were compared between Medicaid and non-Medicaid cohorts using adjusted odds ratios and cost/LOS analyses.	Medicaid payer status was an independent predictor of higher 90-d readmission and reoperation rates, greater likelihood of prolonged hospitalization, and increased direct costs following primary shoulder arthroplasty. The authors highlight the need for risk-adjustment models that incorporate payer status to support equitable access and appropriate reimbursement.
Hartwell^25^ 2019	Total shoulder arthroplasty: Risk factors for a prolonged length of stay. A retrospective cohort study	Single-institution retrospective cohort	The study used a national surgical quality database to compare patients undergoing total shoulder arthroplasty by post-operative length of stay (≤24 h vs. > 24 h). Univariate and multivariate analyses evaluated pre-operative comorbidities, demographic characteristics, and intraoperative variables associated with extended hospitalization.	Several medical comorbidities and higher perioperative risk profiles were independently associated with hospital stays exceeding 24 h, whereas male sex was protective. These findings highlight key patient and operative factors that may inform perioperative optimization and outpatient pathway selection.
Lyman^35^ 2005	The association between hospital volume and total shoulder arthroplasty outcomes	New York State Statewide Planning and Research Cooperative System (SPARCS) database, 1997–2000	Evaluated whether hospital procedure volume was associated with post-operative outcomes following total shoulder arthroplasty using a statewide discharge database. Outcomes assessed included length of stay, hospital costs, short-term readmission, early revision, and short-term mortality. Multivariable analyses adjusted for demographic and clinical factors were used to determine independent relationships between volume and outcomes.	Higher-volume hospitals demonstrated a lower adjusted risk of 60-d readmission, while other outcomes showed no significant association with volume. Findings suggest potential quality advantages linked to institutional experience, though further research is required before informing policy decisions.
Hammond^24^ 2003	Surgeon Experience and Clinical and Economic Outcomes for Shoulder Arthroplasty	New York State Statewide Planning and Research Cooperative System (SPARCS) database	Evaluated whether surgeon case volume was associated with clinical and economic outcomes following total shoulder arthroplasty or hemiarthroplasty, using a statewide hospital discharge database and multivariable adjustment for patient, procedural, and hospital factors.	Higher surgeon volume was associated with lower complication rates and shorter hospital stays, with no significant difference in adjusted costs. Findings support the value of experience and ongoing training in optimizing shoulder arthroplasty outcomes.
Lee^32^ 2020	Preoperative hyponatremia is associated with postoperative major morbidity, prolonged length of stay, non-home discharge, and readmission in total shoulder arthroplasty	ACS-NSQIP (American College of Surgeons National Surgical Quality Improvement Program)	This study used a national surgical quality database to evaluate whether pre-operative hyponatremia independently predicts adverse 30-d outcomes following anatomic or reverse total shoulder arthroplasty. Multivariable logistic regression analyses were performed with stringent statistical correction to assess associations with post-operative morbidity and healthcare utilization metrics.	Pre-operative hyponatremia was an independent predictor of increased short-term morbidity and higher post-operative resource utilization after total shoulder arthroplasty. The authors highlight the need for thorough pre-operative optimization and call for further work to clarify the effect of hyponatremia severity and correction on perioperative outcomes.
Vanderham^66^ 2024	Psychiatric comorbidities are an independent risk factor for perioperative and postoperative complications in patients undergoing primary total shoulder arthroplasty	Nationwide Readmissions Database (NRD)/HCUP, 2016–2020 (same research group as Gammel 2025/Guareschi 2024; NRD confirmed from co-authors' parallel publications)	Evaluated the impact of pre-operative mental health disorders on perioperative adverse events, length of stay, and medical/mechanical complications within 180 d after primary TSA using a nationwide administrative database and multivariable logistic regression.	Pre-operative mental health disorders independently increased the likelihood of perioperative and mechanical complications and revision, indicating a need for heightened perioperative counseling and post-operative surveillance in this population.
Singh^57^ 2014	The effect of surgeon and hospital volume on shoulder arthroplasty perioperative quality metrics	National Inpatient Sample (NIS)/HCUP	Prospectively evaluated registry data to determine whether surgeon and hospital shoulder arthroplasty volume independently influence perioperative metrics across hemiarthroplasty, anatomic total shoulder arthroplasty, and reverse shoulder arthroplasty, using correlation analyses and adjusted covariance models.	Higher surgeon and hospital volumes were associated with more favorable perioperative metrics across all procedure types, with surgeon volume exerting the stronger effect. Findings support centralization of complex shoulder arthroplasty to higher-volume surgeons and centers to optimize operative efficiency.
Koh^31^ 2018	Thirty-day Complications and Readmission Rates in Elderly Patients After Shoulder Arthroplasty	ACS-NSQIP (American College of Surgeons National Surgical Quality Improvement Program), 2006–2015	This study analyzed national registry data to compare 30-d post-operative complications, readmissions, discharge patterns, and operative characteristics across age groups undergoing total shoulder arthroplasty, reverse shoulder arthroplasty, or hemiarthroplasty. Multivariable logistic regression was used to identify independent predictors of short-term complications.	Elderly patients demonstrated markedly higher 30-d complication and unplanned readmission rates. Although age contributed to risk, surgical indication and pre-existing comorbidities were the strongest independent predictors. The authors recommend thorough perioperative counseling and medical optimization for elderly candidates to mitigate risk.
Singh^61^ 2015	Age-related differences in the use of total shoulder arthroplasty over time USE AND OUTCOMES	National Inpatient Sample (NIS)/HCUP	This study analyzed national utilization patterns and age-specific differences in TSA using a large inpatient database over a 12-year period. Trends were evaluated across predefined age groups, and temporal changes in perioperative outcomes were assessed using appropriate trend and regression analyses.	TSA utilization increased substantially over time, with widening age-related disparities in key post-operative outcomes. The evolving epidemiology highlights the need for targeted strategies to optimize care in older patient groups exhibiting less favorable outcome profiles.
Schairer^54^ 2015	National utilization of reverse total shoulder arthroplasty in the United States	National Inpatient Sample (NIS)/HCUP	Used a national inpatient database to characterize utilization patterns and surgical indications for hemiarthroplasty, TSA, and rTSA across the United States. Examined associated diagnoses and hospital factors, and applied multivariable regression to identify determinants of prolonged length of stay.	rTSA represented a substantial proportion of arthroplasty procedures, with notable uptake for proximal humerus fractures in addition to cuff-related pathology. Procedure choice varied by indication, and hospital stay was influenced by patient comorbidity and insurance status.
Yin^71^ 2019	The Effect of Race on Early Perioperative Outcomes After Shoulder Arthroplasty: A Propensity Score Matched Analysis	ACS-NSQIP (American College of Surgeons National Surgical Quality Improvement Program), 2006–2015	Evaluated racial differences in 30-d post-operative outcomes after shoulder arthroplasty using a propensity-matched cohort from a national quality improvement database. Compared post-operative complications and readmissions between Black and White patients using multivariable analyses.	Short-term complication and readmission rates were comparable between racial groups; however, Black patients demonstrated increased operative time, prolonged hospitalization, and higher short-term mortality, highlighting persistent disparities in perioperative outcomes.
Sharma^56^ 2024	Social determinants of health disparities impact postoperative complications in patients undergoing total shoulder arthroplasty	PearlDiver Mariner national all-payer insurance claims database (2015–2019 Q3; minimum 1 yr longitudinal follow-up)	Retrospective cohort study using national insurance claims data to identify SDHDs via ICD coding and compare post-operative medical and surgical complication rates after primary TSA. Patients with and without documented SDHDs were matched 1:1 on demographics and comorbidities before multivariable analysis of short-term medical and midterm surgical outcomes.	A history of SDHDs independently increased the likelihood of post-operative medical complications, prolonged hospitalization, and subsequent revision procedures after TSA, underscoring the need for risk stratification and tailored care pathways in this population.

*BMI*, body mass index; *aTSA*, anatomic total shoulder arthroplasty; *rTSA*, reverse total shoulder arthroplasty; *LOS*, length of stay; *SDHD*, social determinants of health disparities; *HA*, hemiarthroplasty; *SD*, standard deviation; *IQR*, interquartile range; *CI*, confidence interval; *OR*, odds ratio; *IRR*, incidence rate ratio; *ROC*, receiver operating characteristic; *CT*, computed tomography; *HCUP*, Healthcare Cost and Utilization Project; *EMR*, electronic medical record; *ASA-PS*, American Society of Anesthesiologists Physical Status; *CNS*, central nervous system; *ICD*, International Classification of Diseases.

**Table III:** Factors associated with length of stay following shoulder arthroplasty.

Author (yr)	Age	Sex	BMI	Ethnicity	Social factors	Health system and structural factors	Clinical factors
Nazzal^41^ 2025	Age: older patients had higher odds of failing same-d discharge (OR 1.44, *P* = .004)	Sex: female patients had higher odds of failing SDD (OR 2.76, *P* = .001)					Rotator cuff arthropathy patients had higher odds of failing SDD compared with osteoarthritis (OR 3.40, *P* < .001)
Areti^2^ 2025			Morbid obesity (BMI ≥40): 2.23 ± 3.253 d Non-morbid obesity: 1.87 ± 1.887 d Mean difference: +0.361 d (*P* < .001)				
Burns^9^ 2024							Prior surgery group: 2.2 ± 1.1 d No prior surgery: 2.4 ± 1.1 d *P* = .016
Gammel^18^ 2025						Medicaid: 1.9 ± 2.5 d Non-Medicaid: 1.5 ± 2.0 d *P* < .001	
Sohn^63^ 2024							Unmatched cohort SOT: 2.61 ± 2.73 d Non-SOT: 1.90 ± 2.03 d *P* < .001 Propensity-matched cohort SOT: 2.61 ± 2.73 d Non-SOT: 2.02 ± 1.67 d *P* < .001
Raji^50^ 2024	Inpatient LOS increases with age: <65 = 24.0 h, 65–74 = 24.48 h, ≥75 = 26.40 h (*P* < .001)						
Mayfield^37^ 2024							All TSA patients (after matching) OA: 1.52 ± 1.05 d IA: 1.59 ± 1.12 d *P* < .001 aTSA subgroup (after matching) OA: 1.42 ± 0.88 d IA: 1.49 ± 0.94 d *P* = .004 rTSA subgroup (after matching) OA: 1.56 ± 1.12 d IA: 1.63 ± 1.19 d *P* = .002
Gruson^22^ 2023	Age (per yr) IRR 1.012, *P* = .026∗	Female: IRR 1.538, *P* < .001 Male: reference	BMI <30 (non-obese): median LOS 2 d (IQR 1–2), Reference 30–40 (obese): median LOS 2 d (IQR 1–3), IRR 1.157, *P* = .177 ≥40 (morbidly obese): median LOS 2 d (IQR 2–4), IRR 1.316, *P* = .038∗				Hemovac use Yes: IRR 1.392, *P* = .005∗ No: Reference Age-adjusted Charlson Comorbidity Index (ACCI) Continuous variable; not significant in Poisson regression
Puzzitiello^49^ 2022	Age – β = 0.18, 95% CI 0.002–0.03, *P* = .022	Female sex – β = 0.22, 95% CI 0.13–0.48, *P* = .001	BMI – β = 0.04, *P* = .54		Limited health literacy (LHL) – β = 0.14, 95% CI 0.02–0.42, *P* = .035 LHL: 1.84 ± 0.92 d HHL: 1.57 ± 0.58 d Proportion with prolonged LOS (>1 night): LHL: 38.9% HHL: 27.1% *P* = .098	Medicare insurance – β = −0.01, *P* = .93	Depression – β = 0.15, 95% CI 0.04–0.45, *P* = .022 ASA score >2 – β = 0.13, *P* = .07
Burns^8^ 2022			Obesity Affected: 2.4 ± 2.4 d (n = 184) Unaffected: 2.2 ± 1.6 d (n = 1,125) *P* = .158 (not significant)		Smoking Affected: 2.1 ± 1.8 d (n = 146) Unaffected: 2.3 ± 1.7 d (n = 1,171) *P* = .244 (not significant)		Anemia Affected: 5.0 ± 4.0 d (n = 37) Unaffected: 2.2 ± 1.6 d (n = 1,042) *P* < .001 Malnutrition Affected: 3.7 ± 2.8 d (n = 116) Unaffected: 2.2 ± 1.5 d (n = 789) *P* < .001 Uncontrolled Diabetes Affected: 2.8 ± 2.8 d (n = 66) Unaffected: 2.3 ± 1.7 d (n = 1,042) *P* = .019 Opioid use Affected: 2.3 ± 1.7 d (n = 427) Unaffected: 2.2 ± 1.7 d (n = 890) *P* = .407 (not significant)
Lunati^34^ 2021							Patients with pre-operative depression 735/3,209 (22.9%) Patients without depression 3,876/19,414 (20.0%) *P* < .001 (univariate) Depression independently predicts extended LOS ≥3 d OR 1.23 (95% CI 1.12–1.36), *P* < .001
Polisetty^48^ 2021	Younger patients more likely SDD (<24h: 64.7 ± 8.3 vs. 72.6 ± 8.3) *P* < .0001	Sex: Male patients more likely SDD (35.8% vs. 22.0%) *P* = .003					OARA score ≤110: SDD: sensitivity 98% for safe SDD NPV: 98.9% Specificity: 24% Mean OARA score increased significantly with LOS (*P* = .001) OARA score >110: SDD: 2% LOS longer than ≤110 group OARA score ≤59: SDD: 66.7% Less predictive for shoulder arthroplasty than ≤110 threshold (*P* < .0001) ASA-PS ≤2: SDD: 80.4% Not significantly associated with LOS (*P* = .82)
Dacombe^13^ 2020	Median LOS by category: <60 yr → 2 d (IQR 1–3) 60–69 yr → 2 d (IQR 1–3) 70–79 yr → 2 d (IQR 2–4) 79 yr → 4 d (IQR 2–8) Independent predictor in regression: *P* = .033	Median LOS: Male → 2 d (IQR 1–3) Female → 3 d (IQR 2–5) Independent predictor in regression: *P* = .021					Chronic kidney disease (CKD): Independent predictor in regression: *P* = .0008 Congestive cardiac failure (CHF): Independent predictor in regression: *P* = .035 Previous myocardial infarction (MI): Independent predictor in regression: *P* = .030
Menendez^38^ 2019	Age: extended LOS 71 ± 9.5 yr vs. not extended 68 ± 7.9 yr—*P* = .001	Female sex: extended LOS 85% vs. male 53% — *P* < .001	Extended LOS: BMI 32.6 ± 7.5 Not extended LOS: BMI 30.2 ± 5.8 *P* = .004	Marital status: Unmarried: extended LOS 58% Married: extended LOS 26% *P* < .001			Number of patient-reported allergies: 3.3 ± 3.8 vs. 1.6 ± 2.1, *P* < .001 ASA score ≥3: 39% vs. ASA ≤2 17%, *P* < .001 Pre-operative opioid use: 24% vs. 14%, *P* = .013 Diabetes: 28% vs. 9%, *P* < .001 Depression: 39% vs. 21%, *P* < .001 Pre-operative ASES score: 27.8 ± 15.1 vs. 36.2 ± 16.4, *P* < .001 Procedure type: rTSA 85% vs. aTSA 66%, *P* < .001
Biron^5^ 2020	Age ≥70 yr Long LOS mean age: 72.8 yr Short LOS mean age: 67.8 yr *P* < .001	Male sex Short LOS: 51.1% male Long LOS: 31.9% male *P* < .001	BMI Short LOS: 31.2 Long LOS: 31.7 *P* = .062 (not significant)	White race Short LOS: 94.3% Long LOS: 92.2% *P* < .001	Smoking history Short LOS: 12.0% Long LOS: 9.74% No significant association reported	More recent yr of surgery *P* = .001	Diabetes Long LOS: 25.8% Short LOS: 16.3% *P* < .001 COPD Long LOS: 12.6% Short LOS: 6.27% *P* < .001 Dyspnea Long LOS: 11.3% Short LOS: 5.61% *P* = .008 Hypertension Long LOS: 75.7% Short LOS: 64.8% *P* = .008 ASA class >2 Long LOS: 77.04% Short LOS: 50.14% *P* < .001 Congestive heart failure (CHF) Long LOS: 0.99% Short LOS: 0.28% *P* = .008 Steroid use Short LOS: 4.48% Long LOS: 5.77% No significant association reported
Singh^58^ 2020					Income Quartiles: 0–25th percentile: OR 0.84 (95% CI 0.80–0.89) → Significantly lower odds of prolonged LOS compared to highest income quartile 25–50th percentile: OR 0.89 (95% CI 0.85–0.94) → Lower odds of prolonged LOS 50–75th percentile: OR 0.90 (95% CI 0.86–0.94) → Lower odds of prolonged LOS 75–100th percentile (reference category): OR 1.0	Insurance type: Medicare: OR 1.60 (95% CI 1.52–1.69) → Significantly higher odds of prolonged LOS compared to Private insurance Medicaid: OR 1.65 (95% CI 1.45–1.87) → Significantly higher odds of prolonged LOS compared to Private insurance Other insurance: OR 1.43 (95% CI 1.29–1.58) → Higher odds of prolonged LOS Self-Pay: OR 1.55 (95% CI 1.18–2.04) → Higher odds of prolonged LOS Private (reference category): OR 1.0	
Navarro^40^ 2019							OA: 1.96 d ON: 2.37 d 21.1% longer LOS for ON *P* = .05
Sabesan^53^ 2020						Large hospitals had slightly longer LOS (2.34 d vs. 2.28 d) *P* < .01 Teaching hospitals had longer LOS (2.76 d vs. 2.56 d)	
Crook^12^ 2018	Elderly patients had significantly longer LOS Elderly patients ≥80 yr: 3.6 d Non-elderly patients <80 yr: 2.2 d *P* < .0001						
Bovonratwet^7^ 2019	Octogenarians had significantly longer LOS ≥80 yr vs. < 70 yr: 2.4 vs. 1.8 d +0.6 d, *P* < .001 ≥80 yr vs. 70–79 yr: +0.4 d, *P* < .001						
Newman^42^ 2018	Octogenarians had significantly longer LOS (3 vs. 2 d, *P* < .0001)						
Kim^30^ 2018	Age (by ECM comorbidity count): ≥90 yr: 39.1% prolonged LOS 18–29 yr: 9.5% prolonged LOS *P* < .0001	Sex (by ECM comorbidity count): Female: 35.8% prolonged LOS Male: 22.5% prolonged LOS *P* < .0001		Race (by ECM comorbidity count): Black: 39.8% prolonged LOS White: 30% Hispanic: 33.4% Asian: 26.3% Native American: 36.1% Other: 29.4% *P* < .0001		Primary payer (by ECM comorbidity count): Medicare: 33.1% prolonged LOS Medicaid: 37.1% Private insurance: 22% Self-pay: 23.1% No charge: 23.5% Other: 21.9% *P* < .0001	ECM comorbidity count: 0 = 4.4% prolonged LOS 1 = 6.1% 2 = 9.2% ≥3 = 18.2% *P* < .0001 CCI comorbidity count: 0 = 7.3% prolonged LOS 1 = 11.8% 2 = 17.6% ≥3 = 27.5% *P* < .0001
Izquierdo-Fernández^27^ 2018			BMI <30: 5.18 d (SD 1.25) BMI ≥30: 5.83 d (SD 1.42) *P* = .2223 BMI <35: 5.43 d (SD 1.25) BMI ≥35: 6.00 d (SD 1.69) *P* = .3265				
Wong^70^ 2017		Men: 2.32 d Women: 2.58 d *P* = .18					
Ramkumar^51^ 2017						Surgeon volume Low (0–4 surgeries/yr): 2.5 d Medium (5–14 surgeries/yr): 2.3 d High (≥15 surgeries/yr): 1.6 d *P* = .022 Hospital volume Low (0–3 surgeries/yr): 2.4 d Medium (4–14 surgeries/yr): 2.3 d High (≥15 surgeries/yr): 2.0 d *P* = .028	APR risk of mortality index Minor risk: 3.2% prolonged LOS (>5 d) Major/extreme risk: 43% prolonged LOS *P* < .001
Issa^26^ 2016	Age (vs. 0–49): 50–64: Estimate −3.01, *P* = .0177 64–79: Estimate 0.27, *P* = .8469 ≥80: Estimate 19.11 (95% CI 15.62–22.70), *P* < .0001	Female vs. male: estimate 18.71 (95% CI 17.57–19.86), *P* < .0001		Black vs. White: Estimate 10.64 (95% CI 7.35–14.04), *P* < .0001 Hispanic vs. White: Estimate 2.28, *P* = .2003 Other vs. White: Estimate −0.78, *P* = .6560		Insurance: Medicaid vs. Medicare: estimate 0.96, *P* < .0001 Private vs. Medicare: estimate −6.44 (95% CI –7.82, −5.04) Other vs. Medicare: estimate 0.73, *P* = .6560 Hospital volume: Medium vs. low: estimate −6.18 (95% CI –8.63, −3.66), *P* < .0001 High vs. low: estimate −12.08 (95% CI –14.17, −9.94), *P* < .0001	Deyo comorbidity score: 1 vs. 0: Estimate 6.25, *P* < .0001 → LOS 3.5 d 2 vs. 0: Estimate 15.25, *P* < .0001 ≥3 vs. 0: Estimate 31.16, *P* < .0001 → LOS 4.3 d Deyo score 0 → LOS 3.2 d
Weinreb^69^ 2016							Rheumatoid arthritis (RA): RA: mean 2.196 d (SD 0.927), Median 2 (IQR 2–3) Non-RA: mean 2.085 d (SD 0.932), Median 2 (IQR 1–3) *P* < .001
Singh^59^ 2015				LOS > median by race (1998–2011): White: 34.4% (60,678) Black: 44.6% (3,431) By time period (unadjusted all *P* < .001): 1998–2000: White 51.4% vs. Black 62% 2001–2002: White 44.7% vs. Black 53% 2003–2004: White 37.2% vs. Black 52.6% 2005–2006: White 38.1% vs. Black 56.7% 2007–2008: White 35.2% vs. Black 45.8% 2009–2011: White 27.3% vs. Black 34.4% Change over time: White −42%, Black −41%			
Kandil^28^ 2016	Older age: OR per yr 1.03 (95% CI 1.02–1.03), *P* < .001 (higher transfusion risk → longer LOS)	Female vs. male: OR 1.94 (95% CI 1.75–2.16), *P* < .001 (higher transfusion risk → longer LOS)	Obesity: lower transfusion risk, *P* < .001 (potentially shorter LOS)	White patients: OR 1.05 (95% CI 1.03–1.07), *P* < .001 (slightly higher transfusion risk)			Charlson–Deyo comorbidity score: OR 1.12 per point, *P* < .001 Ischemic heart disease: OR 1.24 (95% CI 1.08–1.41), *P* < .001 Deficiency anemia: OR 3.52 (95% CI 3.14–3.96), *P* < .001 (strongest predictor) Coagulation disorders: OR 2.25 (95% CI 1.69–2.99), *P* < .001 Blood loss anemia: OR 1.65 (95% CI 1.21–2.23), *P* < .001
Sivasundaram^62^ 2016	Age (vs. < 64y): 65–74y: OR 2.10 (95% CI 1.65–2.70), *P* < .001 75–84y: OR 5.80 (95% CI 4.51–7.38), *P* < .001 ≥85y: OR 17.9 (95% CI 13.0–24.8), *P* < .001	Female sex (vs. male): OR 2.76 (95% CI 2.38–3.20), *P* < .001			Household income (vs. ≤$39,000): all quartiles NS Region (vs. West): Northeast: OR 1.39, *P* = .006 Midwest: OR 1.23, *P* = .040 South: OR 3.68 (95% CI 3.05–4.44), *P* < .001 Discharge quarter (Jan–Mar vs. Oct–Dec): OR 0.76, *P* = .006 (protective)	Primary payer (vs. private): Medicare: OR 1.99 (95% CI 1.58–2.52), *P* < .001 Medicaid: OR 4.77 (95% CI 2.66–8.58), *P* < .001 Self-pay: OR 0.58, *P* = .080 (NS) Hospital location/teaching status: all NS Hospital bed size: all NS Hospital ownership: all NS	No. of chronic conditions per added condition: OR 1.13, *P* < .001 Total LOS per added d: OR 3.29, *P* < .001 Days from admission to procedure (per d): OR 0.45, *P* < .001 (protective) Procedure type: rTSA OR 1.26, *P* = .001; fracture OR 2.04; revision OR 0.51 (protective) CHF: OR 2.00, *P* < .001 Diabetes mellitus: OR 1.51, *P* < .001 Hypertension: NS; CKD: NS; PVD: NS
Basques^3^ 2015	Age 70–79 vs. 60–69: OR 1.84 (95% CI 1.08–3.15), *P* = .026 Age ≥80 vs. 60–69: OR 3.62 (95% CI 2.10–6.27), *P* < .001 Extended LOS (>3 d): Age 70–79 vs. 60–69: OR 1.71, *P* = .049 Age ≥80 vs. 60–69: OR 3.38, *P* < .001						ASA ≥3: OR 2.03 (95% CI 1.32–3.13), *P* = .001 Diabetes: OR 2.37 (95% CI 1.53–3.66), *P* < .001 Pulmonary disease: OR 1.79 (95% CI 1.06–3.03), *P* = .029 Heart disease: OR 1.88 (95% CI 1.03–3.42), *P* = .037
Matsen^36^ 2015	Age (vs. < 45 yr): 45–54: 1.01× LOS (NS) 55–64: 1.01× LOS (NS) 65–74: 1.04× LOS (NS) 75–84: 1.17× LOS, *P* < .001 >84: 1.38× LOS, *P* < .001 Prolonged LOS (>2 d) TSA >84 yr: 63%; HA >84 yr: 80.2%	Male vs. female (female = reference): LOS 14% shorter in males (0.86×), *P* < .001 Prolonged LOS (>2 d): TSA Female 50.6%, male 28.3%; HA female 64.3%, male 43.5%		African American vs. White: 1.14× LOS, *P* < .001 Other vs. White: 1.06× LOS, *P* < .001		Insurance (vs. commercial/self-insured): Medicaid: 1.14× LOS, *P* < .001 Medicare: 1.04× LOS, *P* < .001	CCI = 1: 1.22× LOS, *P* < .001 CCI >1: 1.73× LOS, *P* < .001 Prolonged LOS (>2 d): CCI = 0 TSA 40.5%; CCI>1 TSA 85.7% Diagnosis effect on LOS: Fracture: 1.12× Osteoarthritis: 0.79× Inflammatory arthritis: 0.78× Cuff tear: 0.80× Osteonecrosis: 0.82×
Singh^60^ 2015						Hospital volume and LOS > median: <5/yr: 46.6% 5–9/yr: 40.4% 10–14/yr: 36.6% 15–24/yr: 34.4% ≥25/yr: 29.2% OR for prolonged LOS vs. < 5/yr: 5–9/yr: OR 0.76, *P* < .0001 10–14/yr: OR 0.67, *P* < .0001 15–24/yr: OR 0.60, *P* < .0001 ≥25/yr: OR 0.47, *P* < .0001	
Pappou^47^ 2014			BMI ≥40 (morbidly obese): 3.1 ± 2.6 d BMI <30: 2.6 ± 1.3 d *P* = .823 (not significant, Mann-Whitney *U* test)				
Griffin^20^ 2014	≤50 yr: 2.94 ± 4.40 d 51–79 yr: 2.85 ± 2.75 d ≥80 yr: 4.00 ± 3.70 d *P* < .001						
Griffin^21^ 2014			Non-obese: 2.56 ± 2.0 d Obese: 2.54 ± 1.67 d Morbidly obese: 2.84 ± 1.77 d *P* = .003				
Khokhar^29^ 2024			Propensity-matched cohorts: Morbidly obese: median 2 d (IQR 1–3) Non-morbidly obese: median 2 d (IQR 2–3) Within-pair difference: 0 (IQR −1 to 1), *P* = .654				
Destine^16^ 2024	Age: OR 0.96 (95% CI 0.90–1.02), *P* = .201	Sex: OR 0.81 (95% CI 0.37–1.78), *P* = .604	BMI: OR 1.03 (95% CI 0.95–1.12), *P* = .413				CCI: OR 1.91 (95% CI 1.39–2.69), *P* < .001 Discharge location: OR 2.52 (95% CI 0.97–6.49), *P* = .056 (NS)
Miltenberg^39^ 2024	Age 61–75 vs. 18–60: OR 1.37 (95% CI 1.06–1.77), *P* = .004 (+37% risk) Age 76+ vs. 18–60: OR 1.98 (95% CI 1.33–2.94), *P* < .001 (+98% risk)	Female vs. male: OR 2.08 (95% CI 1.76–2.45), *P* < .001 (+108% risk)	BMI 25–30 vs. < 25: OR 0.73 (95% CI 0.59–0.91), *P* = .012 (−27% risk) BMI 31–35 vs. < 25: OR 0.72 (95% CI 0.57–0.91), *P* = .012 (−28% risk) BMI 36+ vs. < 25: OR 0.87 (95% CI 0.69–1.11), *P* = .012 (−13% risk, NS)	Black vs. White: OR 1.44 (95% CI 1.00–2.07), *P* < .001 (+44% risk) Asian vs. White: OR 1.35 (95% CI 0.65–2.81), *P* < .001 (+33% risk, NS) Other vs. White: OR 3.21 (95% CI 2.67–3.84), *P* < .001 (+221% risk)	Lives alone vs. with others: OR 1.57 (95% CI 1.18–2.09), *P* = .004 (+57% risk)		Anemia: OR 1.43 (95% CI 1.21–1.69), *P* < .001 ASA grade 3–5: OR 1.88 (95% CI 1.58–2.23), *P* < .001 COPD: OR 1.89 (95% CI 1.44–2.47), *P* < .001 Dependent functional status: OR 3.28 (95% CI 2.24–4.81), *P* < .001 Diabetes mellitus: OR 2.85 (95% CI 1.88–3.72), *P* < .001 Operative time >130 min: OR 2.46 (95% CI 2.08–2.90), *P* < .001 CHF: higher in OHS, significant univariate only Hypertension: significant univariate only Smoking: not significant (*P* = .848) 926/5,811 (15.9%) had OHS >1 d
Guareschi^23^ 2024							Prior bariatric surgery: 1.7 ± 1.8 d No prior bariatric surgery: 1.7 ± 1.6 d *P* = .991
Dawes^15^ 2023						Unmatched: Medicaid 2.89 ± 7.20 d vs. Control 2.38 ± 2.96 d, *P* < .001 Propensity-matched: Medicaid 2.86 ± 7.14 d vs. Control 2.46 ± 3.53 d, *P* < .001	
Hartwell^25^ 2019	LOS ≤24h: mean age 67.4 ± 9.7 yr LOS >24h: mean age 70.3 ± 9.7 yr *P* < .0001	LOS ≤24h: 54.7% male (38.1% female) LOS >24h: 45.3% male (61.9% female) *P* < .0001	BMI: LOS ≤24h: 31.0 ± 6.5 LOS >24h: 31.1 ± 7.1 *P* = .29				
Lyman^35^ 2005						Hospital volume (Kruskal-Wallis *P* < .01 for means): Low (1–15 TSA): Median 3 d; Mean 3.9 ± 3.8 Middle (16–47 TSA): median 3 d; mean 3.1 ± 2.5 High (48+ TSA): median 3 d; mean 3.6 ± 3.1	
Hammond^24^ 2003	Age ≥65: OR 2.2 (95% CI 1.3–3.8) for prolonged LOS >6 d			Black race: OR 2.5 (95% CI 1.3–4.7) for prolonged LOS >6 d	Married: OR 0.4 (95% CI 0.3–0.7) for prolonged LOS >6 d (protective)	High-volume surgeon vs. low: OR 0.3 (95% CI 0.2–0.6) for prolonged LOS >6 d Hemiarthroplasty vs. total: OR 2.3 (95% CI 1.4–3.7)	Charlson ≥1: OR 1.8 (95% CI 1.4–2.4) for prolonged LOS >6 d
Lee^32^ 2020	Age (per yr): OR 1.05 (99% CI 1.04–1.06), significant	Male sex: OR 0.48 (99% CI 0.42–0.55), significant (protective)	BMI (per kg/m^2^): OR 1.01 (99% CI 1.00–1.02), significant	White race: OR 0.72 (99% CI 0.61–0.85), significant (protective)			Hyponatremia (<135 mEq/L): OR 1.63 (99% CI 1.31–2.03) Bleeding disorders: OR 2.58 (99% CI 1.65–4.02) CHF: OR 2.17 (99% CI 1.07–4.39) COPD: OR 1.49 (99% CI 1.19–1.86) Diabetes: OR 1.49 (99% CI 1.28–1.73) Dialysis: OR 3.44 (99% CI 1.61–7.35) Dyspnea: OR 1.37 (99% CI 1.10–1.72) Functional dependence: OR 2.73 (99% CI 2.05–3.64) Sepsis: OR 3.34 (99% CI 1.60–6.96) Smoker: OR 1.20 (NS) Steroid use: OR 1.08 (NS)
Vanderham^66^ 2024							Proportion LOS >2 d: Mental health disorder (MHD): 20% vs. no MHD: 14% Anxiety: 20% Depression: 20% Psychosis: 23% All *P* < .001
Singh^57^ 2014						Surgeon volume and LOS: Low: TSA 1.8, HA 2.0, rTSA 2.3 d, *P* = .01 Medium: TSA 1.9, HA 1.7, rTSA 2.6 d, *P* < .0001 High: TSA 1.7, HA 1.7, rTSA 1.9 d, *P* = .55 Hospital volume and LOS: Low: TSA 1.7, HA 1.9, rTSA 2.4 d, *P* < .0001 Medium: TSA 1.8, HA 1.9, rTSA 2.0 d, *P* = .48 High: TSA 1.9, HA 1.4, rTSA 2.3 d, *P* = .0004	
Koh^31^ 2018	≥80 yr: 2.6 d 65–79 yr: 2.1 d <65 yr: 1.8 d Differences statistically significant (*P* < .001)						
Singh^61^ 2015	Proportion with prolonged LOS by age group: <50 yr: 28.0% 50–64 yr: 25.7% 65–79 yr: 36.1% ≥80 yr: 56.4% *P* < .001 (logistic regression)						
Schairer^54^ 2015	Age ≥75 vs. < 65: OR 1.51 (95% CI 1.15–1.98) for prolonged LOS					Insurance type (vs. private): Medicaid: OR 2.04 (95% CI 1.20–3.46) Other: OR 1.51 (95% CI 1.03–2.20) Hospital characteristics: Teaching hospital vs. non-teaching: OR 1.38 (95% CI 1.17–1.62) High annual rTSA volume (≥24/y): OR 0.60 (95% CI 0.46–0.77) (protective)	Comorbidity burden: 2 comorbidities vs. ≤ 1: OR 1.69 3 comorbidities vs. ≤ 1: OR 2.35 ≥4 comorbidities vs. ≤ 1: OR 5.64 Diagnosis: Humerus fracture: OR 8.49 Procedure type: rTSA vs. TSA: OR 1.96 Hemiarthroplasty vs. TSA: OR 1.55
Yin^71^ 2019				White: 2.1 ± 1.7 d Black: 2.4 ± 2.2 d *P* < .001 (statistically significant)			
Sharma^56^ 2024					Social determinants of health disparity (SDHD): 3.71 ± 19.92 d Control: 2.00 ± 1.28 d *P* < .001		

*BMI*, body mass index; *OA*, osteoarthritis; *ECM*, Elixhauser Comorbidity Measure; *LOS*, length of stay, *ASA*, American Society of Anesthesiologists; *aTSA*, anatomic total shoulder arthroplasty; *rTSA*, reverse total shoulder arthroplasty; *LOS*, length of stay; *SDD*, same-day discharge; *COPD*, chronic obstructive pulmonary disease; *HA*, hemiarthroplasty; *SD*, standard deviation; *IQR*, interquartile range; *CI*, confidence interval; *OR*, odds ratio; *IRR*, incidence rate ratio; *PVD*, peripheral vascular disease; *APR*, all patient refined (risk of mortality index); *NS*, not significant; *ASES*, American Shoulder and Elbow Surgeons (score).

∗ Indicates statistical significance (typically *P* < .05).

The studies were predominantly conducted in the United States (n = 50), with smaller contributions from the United Kingdom (n = 1) and Spain (n = 1). The majority of studies were retrospective cohort or database analyses, with a small number of retrospective cross-sectional studies and prospective registry-based observational studies (Tables I and II).

### Risk of bias

Risk of bias in the 52 non-randomized studies was assessed using ROBINS-I (Fig. 2). Overall, 48.0% of studies were rated moderate and 52.0% serious risk, with none rated low. Confounding was moderate in all studies (100%). Participant selection was moderate in 62.8% and low in 37.2%. Intervention classification was low in 66.7% and moderate in 33.3%, while deviations from intended interventions were low in all studies (100%). Missing data were moderate in 74.5% and low in 25.5%. Outcome measurement was low in 52.9%, moderate in 43.1%, and serious in 2.0%, and selective reporting was moderate in 84% and low in 16%. Confounding, missing data, and selective reporting were the main drivers of elevated risk, explaining the predominance of moderate and serious ratings.

### Patient factors

#### Age

Twenty-six studies evaluated the effect of age on LOS following SA.^3^^,^^5^^,^^7^^,^^12^^,^^13^^,^^16^^,^^20^^,^^22^^,^^24^, ^25^, ^26^^,^^28^^,^^30^, ^31^, ^32^^,^^36^^,^^38^^,^^39^^,^^41^^,^^42^^,^^48^, ^49^, ^50^^,^^54^^,^^61^^,^^62^ Across studies, increasing age was consistently associated with prolonged LOS. Crook et al^12^ reported significantly longer LOS in elderly patients (≥80 years) compared to non-elderly (<80 years): 3.6 vs. 2.2 days, *P* < .0001. Bovonratwet et al^7^ demonstrated octogenarians had longer LOS compared with patients <70 years (2.4 vs. 1.8 days; +0.6 days, *P* < .001) and 70-79 years (+0.4 days, *P* < .001). Similarly, Newman et al^42^ found mean LOS of 3 ± 3.3 days in patients ≥80 versus 2 ± 2.8 days in those <80 (*P* < .0001).

Regression analyses further quantified this relationship. Issa et al^26^ reported relative to patients aged 0-49 years, LOS estimates were significantly higher in patients ≥80 years (estimate 19.11, 95% confidence interval [CI]: 15.62-22.70, *P* < .0001), whereas younger age categories did not show significant differences. Basques et al^3^ demonstrated odds of prolonged LOS increased with age: 70-79 vs. 60-69 years, odds ratio (OR) 1.84 (95% CI: 1.08-3.15, *P* = .026); ≥80 vs. 60-69 years, OR 3.62 (95% CI: 2.10-6.27, *P* < .001). Griffin et al^20^ observed mean LOS of 4.00 ± 3.70 days in patients ≥80, compared with 2.85 ± 2.75 days in 51-79 years and 2.94 ± 4.40 days in ≤50 years (*P* < .001). Koh et al^31^ reported LOS of 2.6 days in ≥80 years, 2.1 days in 65-79 years, and 1.8 days in <65 years (*P* < .001).

Additional studies reinforced the positive correlation between age and LOS. Nazzal et al^41^ found older patients had higher odds of failing same-day discharge (OR: 1.44, *P* = .004). Raji et al^50^ demonstrated increasing LOS with age (<65 = 24.0 hours, 65-74 = 24.48 hours, ≥75 = 26.40 hours, *P* < .001). Gruson et al^22^ quantified that each additional year of age increased expected LOS by 1.2% (incidence rate ratio [IRR] 1.012, *P* = .026). Other studies reported similar trends, including Puzzitiello et al^49^ (β = 0.18, 95% CI: 0.002-0.03, *P* = .022), Menendez et al^38^ (extended LOS: 71 ± 9.5 vs. 68 ± 7.9 years, *P* = .001), and Kim et al^30^ (≥90 years: 39.1% prolonged LOS vs. 9.5% in 18-29 years, *P* < .0001).

Some studies did not demonstrate a significant association between age and LOS. Destine et al^16^ analyzed age as a continuous variable in multivariable logistic regression (OR: 0.96, 95% CI: 0.90-1.02, *P* = .201), and a minority showed non-significant differences for intermediate age groups (Matsen et al,^36^ 45-74 years). Overall, age ≥75 years consistently emerged as a robust predictor of prolonged hospitalization.

#### Sex

Seventeen studies evaluated the effect of sex on LOS.^5^^,^^13^^,^^16^^,^^22^^,^^25^^,^^26^^,^^28^^,^^30^^,^^32^^,^^36^^,^^38^^,^^39^^,^^41^^,^^48^^,^^49^^,^^62^^,^^70^ Female sex was generally associated with prolonged LOS. Issa et al^26^ reported an estimate of 18.71 (95% CI: 17.57-19.86, *P* < .0001) for females versus males. Nazzal et al^41^ found female sex significantly increased odds of failing same-day discharge (OR: 2.76, *P* = .001). Gruson et al^22^ reported IRR 1.538 for female patients (*P* < .001). Puzzitiello et al^49^ found β = 0.22 (95% CI: 0.13-0.48, *P* = .001).

Polisetty et al^48^ observed higher same-day discharge rates in males (35.8% vs. 22.0%, *P* = .003), while Dacombe et al^13^ reported median LOS of 2 days in males vs. 3 days in females (regression *P* = .021). Menendez et al^38^ noted prolonged LOS in 85% of females compared to 53% of males (*P* < .001). Several additional studies confirmed female sex as an independent risk factor, with consistent statistical significance (*P* < .001).^25^^,^^28^^,^^30^^,^^32^^,^^36^^,^^39^^,^^62^

Wong et al^70^ and Destine et al^16^ were notable exceptions, reporting non-significant differences between sexes (Wong et al^70^: 2.32 vs. 2.58 days, *P* = .18; Destine et al^16^: OR: 0.81, 95% CI: 0.37-1.78, *P* = .604).

### Body mass index

Twelve studies reported associations between BMI and LOS.^2^^,^^5^^,^^8^^,^^16^^,^^21^^,^^22^^,^^25^^,^^27^^,^^29^^,^^32^^,^^38^^,^^47^ Morbid obesity (BMI ≥40) was consistently associated with longer LOS. Areti et al^2^ reported 2.23 ± 3.25 days vs. 1.87 ± 1.89 days in non-morbidly obese patients (*P* < .001). Griffin et al^21^ observed a significant difference across BMI categories (non-obese: 2.56 ± 2.0 days, obese: 2.54 ± 1.67 days, morbidly obese: 2.84 ± 1.77 days, *P* = .003). Gruson et al^22^ demonstrated an IRR of 1.316 for BMI ≥40 (*P* = .038).

Other studies found minimal or non-significant effects of BMI on LOS.^5^^,^^8^^,^^16^^,^^25^^,^^27^^,^^29^^,^^47^ Lee et al^32^ reported a small association per kg/m^2^ (OR: 1.01, 99% CI: 1.00-1.02). Overall, extreme obesity may modestly prolong LOS, whereas mild to moderate BMI variations appear less impactful.

### Ethnicity

Nine studies stratified by ethnic background.^5^^,^^24^^,^^26^^,^^28^^,^^30^^,^^36^^,^^39^^,^^59^^,^^71^ Patients of Black ethnicities were found to have longer LOS in multiple analyses. Issa et al^26^ reported Black patients had significantly longer LOS than White patients (estimate 10.64, 95% CI: 7.35-14.04, *P* < .0001). Singh et al^59^ demonstrated higher proportions of LOS > median in Black vs. White patients across multiple time periods (44.6% vs. 34.4%; *P* = .02 unadjusted). Yin et al^71^ reported 2.4 ± 2.2 days vs. 2.1 ± 1.7 days in Black vs. White patients (*P* < .001). Matsen et al^36^ reported African American patients had a 1.14× LOS increase (*P* < .001). Miltenberg et al^39^ reported OR 1.44 for Black vs. White (*P* < .001). Other racial groups (Asian, Hispanic, Native American, and other) showed variable associations, with some studies demonstrating modest increases in LOS.^30^^,^^39^

### Social factors

Seven studies reported social determinants of health.^24^^,^^38^^,^^39^^,^^49^^,^^56^^,^^58^^,^^62^ Sharma et al^56^ found the presence of social determinants of health disparities was associated with longer LOS (3.71 ± 19.92 vs. 2.00 ± 1.28 days, *P* < .001).Singh et al^58^ observed significantly lower odds of prolonged LOS in the lower-income quartiles compared with the highest income quartile (0-25th percentile: OR: 0.84, 95% CI: 0.80-0.89; 25-50th percentile: OR: 0.89, 95% CI: 0.85-0.94; 50-75th percentile: OR: 0.90, 95% CI: 0.86-0.94). Sivasundaram et al^62^ found no significant association between household income and LOS.

Home support and marital status were influential: Miltenberg et al^39^ reported living alone increased odds of prolonged LOS by 57% (OR 1.57, *P* = .004), while Hammond et al^24^ and Menendez et al^38^ demonstrated protective effects of being married (OR: 0.4; prolonged LOS: unmarried 58% vs. married 26%, *P* < .001). Limited health literacy was associated with longer LOS in Puzzitiello et al^49^, with an absolute difference of 0.27 days (6.5 hours), highlighting a potentially modifiable pre-operative factor.

### Health system and structural factors

#### Insurance

Eight studies assessed insurance status.^15^^,^^18^^,^^26^^,^^30^^,^^36^^,^^54^^,^^58^^,^^62^ Medicaid coverage was consistently associated with longer LOS. Gammel et al^18^ reported LOS of 1.9 ± 2.5 vs. 1.5 ± 2.0 days for Medicaid vs. non-Medicaid patients (*P* < .001). Singh et al^58^ reported higher odds of prolonged LOS for Medicare (OR: 1.60, 95% CI: 1.52-1.69), Medicaid (OR: 1.65, 95% CI: 1.45-1.87), and other insurance types (OR: 1.43, 95% CI: 1.29-1.58) compared with private insurance. Kim et al^30^ demonstrated prolonged LOS rates of 37.1% for Medicaid, 33.1% for Medicare, and 22% for private insurance (*P* < .0001). Sivasundaram et al corroborated these findings, with Medicaid OR 4.77 (*P* < .001).^62^ Matsen et al^36^ and Schairer et al^54^ also reported longer LOS in Medicaid and Medicare patients.

#### Hospital and surgeon volume

Seven studies assessed hospital or surgeon volume.^35^^,^^51^^,^^53^^,^^54^^,^^57^^,^^60^^,^^62^ Higher surgical volume was generally protective against prolonged LOS. Sabesan et al^53^ reported slightly longer LOS in large hospitals (2.34 vs. 2.28 days, *P* < .01). Lyman et al^35^ and Singh et al^60^ demonstrated shorter LOS in high-volume hospitals and with high-volume surgeons.^,^ Ramkumar et al^51^ reported low-volume surgeons (0-4 surgeries/yr) had LOS 2.5 days vs. 1.6 days in high-volume surgeons (≥15/yr, *P* = .022); low-volume hospitals (0-3/yr) had LOS 2.4 days vs. 2.0 days in high-volume hospitals (≥15/y, *P* = .028). Teaching hospitals were associated with longer LOS (Schairer et al^54^: OR: 1.38), whereas high annual rTSA volume was protective (OR: 0.60).

### Clinical factors

#### Indication for surgery

Four studies evaluated surgical indication.^36^^,^^37^^,^^40^^,^^69^ Osteoarthritis (OA) was generally associated with shorter LOS compared with osteonecrosis or fracture. Mayfield et al^37^ reported TSA OA: 1.52 ± 1.05 days vs. inflammatory arthritis 1.59 ± 1.12 days (*P* < .001). Navarro et al^40^ reported an LOS of 1.96 vs. 2.37 days for OA vs. osteonecrosis (*P* = .05). Matsen et al^36^ found fractures associated with prolonged LOS (TSA 68%, hemiarthroplasty 71.6%), whereas OA and inflammatory arthritis were associated with shorter LOS. Rheumatoid arthritis was associated with marginally longer LOS (Weinreb et al^69^: 2.196 vs. 2.085 days, *P* < .001).

#### Prior surgery

Three studies assessed prior surgeries.^9^^,^^23^^,^^63^ Burns et al^9^ reported slightly shorter LOS in patients with prior surgery (2.2 ± 1.1 vs. 2.4 ± 1.1 days, *P* = .016). Sohn et al ^63^ found solid organ transplant recipients had significantly longer LOS (2.61 ± 2.73 vs. 1.90 ± 2.03 days, *P* < .001). Guareschi et al^23^ reported no difference in LOS after prior bariatric surgery.

## Discussion

### Principle findings

This systematic review, including over 2.1 million patients, identifies a consistent set of predictors for prolonged hospital stay after SA. Advancing age (≥75-80 years) and female sex were the strongest and most reproducible risk factors, with analyses showing LOS increases of up to 1.8 days and substantially higher odds of failing same-day discharge. Morbid obesity (BMI ≥40 kg/m^2^), non-White ethnicity particularly Black patients, adverse social determinants (living alone, unmarried status, limited health literacy), public insurance (Medicaid or Medicare), low hospital or surgeon volume, and non-elective indications (fracture or inflammatory arthritis) were also independently associated with longer stays. In contrast, milder obesity and prior ipsilateral surgery had minimal or inconsistent effects. Overall, certainty of evidence was highest for age, sex, and provider volume, moderate for BMI, insurance status, and surgical indication, and lower for ethnicity and social determinants due to residual confounding and variability in adjustment across studies. Collectively, these findings reflect a multifactorial set of determinants in which non-modifiable demographic factors interact with modifiable social and structural influences, providing a robust framework for risk stratification and perioperative planning.

### Clinical significance

This review identifies consistent patient- and system-level factors associated with prolonged LOS after SA, with direct implications for outpatient and same-day discharge pathways. Advanced age (≥75 years), female sex, public insurance, low hospital or surgeon volume, and limited social support were associated with longer hospital stay, and increased likelihood of failed same-day discharge, with effects of up to approximately 1-2 days. These variables should be interpreted as risk stratifiers rather than exclusion criteria and may inform targeted perioperative optimization including pre-operative medical assessment, structured discharge planning, and early identification of patients with limited home support. System-level factors, particularly procedural volume and insurance status, further highlight the role of institutional resources and healthcare access in discharge outcomes, supporting the potential value of centralization to higher-volume centers and standardized outpatient pathways where feasible. Overall, these findings support a structured risk-stratified approach to outpatient SA that integrates patient- and system-level variables to improve safety, efficiency, and consistency of care.

### Evaluation in relation to other studies

Berman et al^4^ conducted a systematic review on the predictors of LOS and discharge disposition after SA, analyzing 22 studies up to November 2017. They identified advancing age (>65-85 years; OR: 1.38-3.69 for extended LOS), female sex (OR: 1.16-2.63), obesity (BMI ≥40 kg/m^2^; OR: 1.97), comorbidities (diabetes, chronic obstructive pulmonary disease, heart/renal disease), rTSA (OR: 1.9), and low hospital/surgeon volume as risk factors, with higher volume protective (OR: 0.45-0.79); they also examined intraoperative factors (eg, nerve blocks OR: 0.89; liposomal bupivacaine reducing LOS by 1 day), which were excluded from the current review due to our focus on patient- and health system-related predictors.^4^ The present review advances this earlier work by including 52 studies and over 2.1 million procedures up to July 2025, providing far greater evidential depth. For shared themes, the current synthesis draws on substantially more studies reporting each predictor: age (26 vs. 5 studies), female sex (17 vs. 3), morbid obesity (12 vs. 3), and provider volume (7 vs. 3), yielding more consistent and robust evidence reflective of contemporary practice.^4^ It further extends the evidence base by incorporating predictors absent from Berman et al^4^, including race, social determinants, and public insurance. These expansions not only strengthen the validity of previously established risk factors but also uncover modifiable system-level contributors, offering novel targets for intervention, such as pre-operative social support planning, insurance navigation, and targeted outpatient protocols, which could meaningfully reduce disparities and optimize perioperative resource allocation.

Comparisons with lower extremity arthroplasty show consistent patterns. Shah et al^55^ (2019), in a meta-analysis of 68 studies and over 21 million total knee arthroplasties, found similar predictors of prolonged LOS: age >70 years (mean difference [MD]: 0.81 days), female gender (MD: 0.32 days), obesity (BMI >30; MD: 0.09 days), non-White race (MD: 0.20 days), higher American Society of Anesthesiologists (ASA) score (MD: 1.12 days), Charlson Comorbidity Index >0 (MD: 0.77 days), and pre-operative anemia (MD 0.66 days). These parallels across upper and lower limb arthroplasty indicate shared demographic and system-level influences on post-operative hospital LOS. The consistency of associated predictors across anatomically distinct joints supports the broader applicability of risk-stratified perioperative approaches developed for SA. Moreover, this alignment suggests that integrated, multidisciplinary risk assessment frameworks may be applied across arthroplasty services to promote more consistent patient selection, pre-operative optimization, and targeted strategies to reduce hospital LOS.

### Contextual interpretation and implications of modifiable and non-modifiable factors

The predictors identified should be interpreted within a holistic clinical context and used alongside clinical judgment when assessing suitability for day-case or short-stay pathways. Demographic variables such as race in isolation are poor surrogate markers for intrinsic risk, definitions of “Black” and ethnicities other than Caucasians varied across studies and often lacked adjustment for key confounders including comorbidities, ASA status, insurance, and social support. In elective colorectal surgery, structured enhanced recovery after surgery (ERAS) pathways reduced overall post-operative LOS (median 5.7 days vs. 8 days, *P* < .01) and eliminated pre-existing differences in LOS between Black and White patients that existed in pre-ERAS cohorts (mean 2.7 days longer for Black patients, *P* < .05).^68^ Similarly, even within ERAS frameworks, patients with low socioeconomic status had longer median LOS (4 vs. 3 days, *P* < .001) and higher odds of infectious (OR: 2.46) and overall complications (OR: 1.50), with Black patients demonstrating longer LOS (5 vs. 3-4 days, *P* = .002).^1^ This evidence supports that structural and process-related factors rather than race per se are primary drivers of LOS variation, and risk stratification should integrate comorbidity burden, functional status, social support, and perioperative optimization alongside demographic factors to avoid stereotyping and ensure equitable decision-making. These predictors should be validated prospectively across diverse, multicenter cohorts to ensure generalizability and safe application in clinical decision-making.

### Health system levers to optimize length of stay

Insurance type and provider volume emerged as actionable system-level determinants with a consistent effect on LOS. Medicaid and Medicare coverage were associated with 0.4-1.5 days longer LOS relative to private insurance, reflecting differences in access to pre-operative optimization, post-operative support, and discharge planning resources. Low hospital or surgeon volume also increased LOS by 0.3-0.9 days, highlighting the protective effect of procedural experience and institutional infrastructure. These system-level insights provide clear targets for intervention: directing higher-risk patients to high-volume centers, and integrating structured discharge planning can mitigate variability in LOS while addressing disparities related to insurance and social support. Supporting the feasibility of data-driven approaches, O'Malley et al^45^ analyzed 40,877 SA procedures from the UK National Joint Registry and developed a predictive tool for same-day discharge using patient demographics, comorbidities (eg, dementia, paraplegia), ASA score, hospital caseload, day of surgery, and implant type. The model demonstrated modest discriminatory performance (area under the curve 0.65; goodness-of-fit 0.82), which likely reflects inherent limitations of registry-based data, including limited granularity regarding functional status, social support, perioperative care pathways, and institutional practices.^45^ Nonetheless, even models with moderate discrimination may offer clinically meaningful support at the population level, illustrating how predictive tools can serve as adjuncts to clinical judgment for identifying candidates for outpatient or short-stay pathways. Future prospective studies incorporating more granular patient-, social-, and system-level variables may further refine and enhance the predictive accuracy of such a model.

### Limitations

Several limitations warrant consideration. All included studies were non-randomized, with ROBINS-I assessments revealing universal moderate confounding and predominant moderate-to-serious overall risk, driven by incomplete adjustment for comorbidities, surgical complexity, and perioperative protocols. Heterogeneity in LOS definitions, including continuous measures in days and dichotomized prolonged stay outcomes, as well as differences in arthroplasty type, including anatomic and reverse procedures, precluded meta-analysis, limiting quantitative pooling of effect sizes. Most studies originated from the United States, this may limit generalizability to healthcare systems with differing reimbursement models, discharge pathways, and availability of post-acute care, where determinants of LOS may vary. Residual publication bias cannot be excluded, and granular data on enhanced recovery protocols and discharge barriers were infrequently reported, constraining insight into potentially modifiable pathways.

### Future directions

Future prospective multicenter studies including non-US healthcare systems and using standardized enhanced recovery protocols are required to validate whether the identified predictors of LOS are generalizable across differing models of care, particularly in publicly funded systems with alternative discharge pathways and social care structures, and to further examine these factors as candidate markers of LOS following SA. Such studies would enable refinement of effect estimates while accounting for variation in perioperative pathways and discharge practices. The consistent predictors identified in this study could subsequently be incorporated into the development and external validation of predictive models or nomograms to support risk stratification and patient selection for outpatient or short-stay pathways. These tools may facilitate more consistent, data-informed perioperative planning and improve alignment between patient risk profiles and post-operative care pathways.

## Conclusion

This review establishes advancing age, female sex, morbid obesity, ethnicities other than Caucasians, adverse social determinants, public insurance, and low provider volume as consistent predictors of prolonged hospital LOS. These findings provide an evidence-based framework for refined patient selection, targeted perioperative optimization, and health system redesign to support safe LOS reduction amid rapidly growing procedural volume. Implementation of risk-stratified pathways informed by these predictors offers substantial potential to enhance value-based care delivery while addressing persistent disparities in resource utilization following SA.

## Disclaimers:

Funding: No funding was disclosed by the authors.

Conflicts of interest: The authors, their immediate families, and any research foundations with which they are affiliated have not received any financial payments or other benefits from any commercial entity related to the subject of this article.
