# Supplementary material for: Determinants of prolonged hospitalization following shoulder arthroplasty: a systematic review of patient and health system factors
Source: JSES Rev Rep Tech. 2026 Aug 26;6(4):100856. doi: 10.1016/j.xrrt.2026.100856 (PMC13634513; doi:10.1016/j.xrrt.2026.100856)
Supplement: Supplementary file 1 [file mmc1.docx]

| **PICO Element** | **Inclusion Criteria** | **Exclusion Criteria** |
| --- | --- | --- |
| **Population (P)** | • Adults (≥18 years) undergoing shoulder arthroplasty: TSA, RSA, or HA • Primary or revision procedures | • Paediatric patients (<18 years) • Non-human or cadaveric studies • Non-arthroplasty shoulder procedures (e.g., arthroscopy) |
| **Intervention / Exposure (I)** | • Patient-related factors (age, sex, BMI, race, comorbidities, prior surgeries, social support) • Health system factors (insurance type, hospital/surgeon volume) | • Studies not assessing determinants or predictors of LOS • Interventions unrelated to LOS (e.g., surgical technique without LOS outcome) |
| **Comparator (C)** | • Patients without the factor of interest or standard reference groups (e.g., younger vs older, private vs public insurance, high- vs low-volume hospitals) | • Studies without comparator/reference group • Studies reporting LOS only without stratification by risk factors |
| **Outcome (O)** | • Length of hospital stay (mean/median LOS) • Prolonged hospitalisation or extended LOS • Odds or risk of failing same-day discharge | • Studies not reporting LOS or prolonged hospitalisation • Studies reporting only complications, functional outcomes, or PROMs without LOS data |
| **Study Design** | • Observational studies: cohort (prospective/retrospective), case-control, cross-sectional • Registry or database studies assessing LOS determinants | • Case reports, reviews, editorials, letters • Conference abstracts without full text |
| **Language** | • English | • Non-English |

Supplementary Table 2 – Inclusion and Exclusion Criteria
