# Supplementary material for: Determinants of prolonged hospitalization following shoulder arthroplasty: a systematic review of patient and health system factors
Source: JSES Rev Rep Tech. 2026 Aug 26;6(4):100856. doi: 10.1016/j.xrrt.2026.100856 (PMC13634513; doi:10.1016/j.xrrt.2026.100856)
Supplement: Supplementary file 2 [file mmc2.docx]

Search String

(((length OR postoperative) AND Stay) OR "Length of Stay" OR "Postoperative Stay" OR LOS OR Hospital Stay) AND ((Shoulder OR "Total shoulder") AND (arthroplasty OR Replacement)) AND (preoperative predictors OR risk factors OR predictive factors OR Predict OR prognosis OR prognostic)

Supplementary Table 1 – Search String
